# Comparison of five Boosting-based models for estimating daily reference evapotranspiration with limited meteorological variables

**DOI:** 10.1371/journal.pone.0235324

**Published:** 2020-06-29

**Authors:** Tianao Wu, Wei Zhang, Xiyun Jiao, Weihua Guo, Yousef Alhaj Hamoud

**Affiliations:** 1 College of Agricultural Engineering, Hohai University, Nanjing, China; 2 State Key Laboratory of Hydrology-Water Resources and Hydraulic Engineering, Hohai University, Nanjing, China; 3 Cooperative Innovation Center for Water Safety & Hydro Science, Hohai University, Nanjing, China; Hellenic Agricultural Organization - Demeter, GREECE

## Abstract

Accurate ET_0_ estimation is of great significance in effective agricultural water management and realizing future intelligent irrigation. This study compares the performance of five Boosting-based models, including Adaptive Boosting(ADA), Gradient Boosting Decision Tree(GBDT), Extreme Gradient Boosting(XGB), Light Gradient Boosting Decision Machine(LGB) and Gradient boosting with categorical features support(CAT), for estimating daily ET_0_ across 10 stations in the eastern monsoon zone of China. Six different input combinations and 10-fold cross validation method were considered for fully evaluating model accuracy and stability under the condition of limited meteorological variables input. Meanwhile, path analysis was used to analyze the effect of meteorological variables on daily ET_0_ and their contribution to the estimation results. The results indicated that CAT models could achieve the highest accuracy (with global average RMSE of 0.5667 mm d^-1^, MAE of 4199 mm d^-1^and Adj_R^2^ of 0.8514) and best stability regardless of input combination and stations. Among the inputted meteorological variables, solar radiation(Rs) offers the largest contribution (with average value of 0.7703) to the R^2^ value of the estimation results and its direct effect on ET_0_ increases (ranging 0.8654 to 0.9090) as the station’s latitude goes down, while maximum temperature (T_max_) showes the contrary trend (ranging from 0.8598 to 0.5268). These results could help to optimize and simplify the variables contained in input combinations. The comparison between models based on the number of the day in a year (J) and extraterrestrial radiation (Ra) manifested that both J and Ra could improve the modeling accuracy and the improvement increased with the station’s latitudes. However, models with J could achieve better accuracy than those with Ra. In conclusion, CAT models can be most recommended for estimating ET_0_ and input variable J can be promoted to improve model performance with limited meteorological variables in the eastern monsoon zone of China.

## Introduction

Reference evapotranspiration (ET_0_) is an essential factor in both of hydrological and ecological process [1–5]. Since ET_0_ plays a crucial role in calculating crop water requirement, water budgeting and agricultural water management, accurate estimation of ET_0_ is very meaningful and also serves as the foundation of realizing water-saving irrigation and intelligent irrigation. Methods of obtaining ET_0_ can be generally divided into three types: experimental method, empirical models and numerical simulations. Although experimental determination can measure ET_0_ directly, it can hardly be popularized due to its tedious operation steps and strong regional limitations [6–8]. Now days, FAO-56 Penman-Monteith (FAO-56 PM) model is generally regarded as the most authentic method for estimating ET_0_ in semiarid and humid regions and the estimation result is also widely used as the target to validate other models in areas where ET_0_ data are not available [9–12]. However, the meteorological variables required by FAO-56 PM model for estimating ET_0_ are difficult to obtain or fully available in most regions, which makes it difficult to be implemented. According to the principle of selecting ideal model for estimating ET_0_ proposed by Shih [13], ideal models should be based on minimal input variables with acceptable accuracy. Therefore, empirical models based on less meteorological variables have evolved to enhance the practicality of empirical models over the years [12,14–16], which can be generally classified as temperature-based, radiation-based, pan evaporation-based, mass transfer-based and combination type [4]. Among all these empirical models, Hargreaves-Samani model [17] requires the least meteorological variables input and has already been proved its accuracy around the world, which makes it the most popular empirical model. Other empirical models based on simplified Penman-Monteith model and solar radiation, such as Priestley-Taylor model [18], Irmak model [19] and Makkink model [20], have also been implemented in areas where full meteorological factors can hardly be obtained. However, these methods usually have such regional limitation and poor portability that they are not suitable to be applied for accurate estimation directly without taking localization approach.

By introducing intelligent algorithms for analyzing the non-linear relationship between meteorological variables and ET_0_, numerical simulation method using machine learning and deep learning have been advanced greatly. Since Kuma first investigated artificial neural network (ANN) models for estimating ET_0_ [21], this kind of method has attracted more and more researchers because of its short time, high precision and strong generalization ability. These algorithms can be generally classified as artificial neural networks-based [9,22–25], tree-based [7,26,27], kernel-based [28,29], heuristic-based [27,30,31] and hybrid algorithm-based [32,33].

To further improve the accuracy of machine learning algorithm in ET_0_ estimation, ensemble learning has drawn attention from more and more researchers. The core idea of ensemble learning is to combine several ‘weak learners’ to build a new ‘strong learner’, so as to reduce bias, variance and improve prediction results. Common ensemble learning models like Random Forest [34], Gradient Boosting Decision Tree [35] and Extreme Gradient Boosting models [36] have already widely used in various classification and regression problems [3,37–39] based on the characteristics of simple structure and high accuracy.

This study provides a comparison of five Boosting-based models to find out the best Boosting-based for estimating daily ET_0_ under the condition of limited input variables in the eastern monsoon zone of China. Therefore, the main purpose of this study produced as follows: (1) to compare the accuracy and stability of Boosting-based models with various input combinations across different climate zones; (2) to find an effective approach for improving the modeling accuracy under the condition of limited input variables.

## Material and methods

### Study area and data description

Geographically, the eastern monsoon zone of China is located in the east of the Great Khingan Mountains, south of the Inner Mongolia Plateau and east of the eastern edge of the Tibetan Plateau, including the second-level Loess Plateau, Sichuan Basin, Yunnan-Guizhou Plateau and the Hengduan Mountain area, as well as the third-level coastal plain and hilly areas. The climate types of the eastern monsoon zone include temperate monsoon climate, subtropical monsoon climate and tropical monsoon climate. This study area is significantly affected by the ocean monsoon in summer and the cold air flow from the north in winter. The annual average temperature changes significantly with latitude, showing a decreasing trend from south to north. This zone accounts for about 45% of the country's land area and 95% of the Chinese total population. As the eastern monsoon zone servers as one of the main farming areas of China, the research on the estimation model of ET_0_ can provide scientific basis for the accurate prediction of crop water demand in this region and improve the utilization efficiency of agricultural water resources, which is of great significance to the sustainable utilization of water resources.

According to the climate type and latitude distribution range of the eastern monsoon zone, 10 meteorological stations (Harbin, Shenyang, Yan 'an, Jinan, Nanjing, Changsha, Chengdu, Kunming, Nanning and Guangzhou) were selected as research stations. To be more specific, Harbin, Shenyang, Yan 'an, Jinan belong to the temperate monsoon zone (TMZ), Nanjing, Changsha, Chengdu, Kunming belong to the subtropical monsoon zone (SMZ) and Nanning, Guangzhou belong to the tropical monsoon zone (TPMZ).

In order to test and verify the accuracy and stability of Boosting-based models for ET_0_ estimation, daily meteorological variables, including maximum(T_max_), and minimum(T_min_) air temperature, relative humidity (RH), wind speed at 2 m height (U_2_) and solar radiation (Rs) from 1997 to 2016 continuously, were selected as the training and testing data set. The above meteorological data was obtained from the National Meteorological Information Center (NMIC) of China Meteorological Administration (CMA) with good quality and high precision and the missing data was interpolated through PYTHON KNN interpolation method in data pre-processing. The annual average values of the main meteorological variables at above stations during the study period were illustrated in Table 1.

**Table 1:** The annual average of the main meteorological variables of 10 stations during the study period.

Climate zone	Station	Longitude	Latitude	Altitude	T_max_	T_min_	U_2_	RH	Rs	P_r_
		(E)	(N)	(m)	(°C)	(°C)	(m s^-1^)	(%)	(MJ m^-2^ d^-1^)	(mm yr^-1^)
TMZ	Harbin	126.5	45.8	165.5	10.0	-1.6	2.8	65.7	13.9	535.0
	Shenyang	123.5	41.7	74.8	14.5	2.4	2.9	61.8	14.6	621.1
	Yan‘an	109.5	36.6	1275.8	16.5	3.4	2.6	53.1	15.4	497.2
	Ji’nan	116.7	36.6	95.6	20.1	7.9	2.5	58.6	15.1	650.4
SMZ	Nanjing	118.8	32.1	25.5	21.5	10.9	2.6	73.0	13.8	1090.7
	Changsha	112.9	28.2	90.4	22.7	13.2	2.1	77.6	11.9	1465.3
	Chengdu	104.1	30.6	617.2	22.2	12.1	1.6	68.7	11.7	939.6
	Kunming	102.8	24.9	1938.5	22.4	10.3	2.5	68.6	16.3	836.1
TPMZ	Nanning	108.4	22.8	160.4	27.1	17.4	2.5	76.4	13.4	1373.1
	Guangzhou	113.3	23.1	64.3	27.1	17.8	2.3	77.3	13.5	1776.7

Where P_r_ is annual average precipitation.

All daily meteorological data were normalized to fall between 0 and 1 to improve the convergence rate of the model and minimize the influence of absolute scale. The normalization equation is as follows [3,24,26]:

$$X_{\text{norm}}\text{=}\frac{X_{\text{0}}\text{-}X_{\text{min}}}{X_{\text{max}}\text{-}X_{\text{min}}}$$ (1)

Where *X*_*norm*_ is the normalized value, *X*_0_, *X*_*min*_, and *X*_*max*_ are the real value, the minimum value, and the maximum value of the same variable, respectively.

### FAO-56 Penman-Monteith model

Since it is difficult to obtain the practical ET_0_ data in this study area, ET_0_ values calculated by the FAO-56 Penman-Monteith model are regarded as the target for training and testing the Boosting-based models, which is a widely used and acceptable practice in this case [2,8,22,30,40].

The FAO-56 PM model is expressed as:

$$ET_{\text{0}}\text{=}\frac{\text{0}.\text{408}\Delta \left(R_{\text{n}}-G\right)+\gamma \frac{900}{T_{mean}+273}U_{2}\left(e_{s}-e_{a}\right)}{\Delta +\gamma \left(1+0.34U_{2}\right)}$$ (2)

Where ET_0_ is reference evapotranspiration (mm d^-1^), *R*_*n*_ is net radiation (MJ m^-2^ d^-1^), G is soil heat flux density (MJ m^-2^ d^-1^), T_mean_ is mean air temperature at 2 m height (°C), *e*_*s*_ is saturation vapor pressure (kPa), *e*_*a*_ is actual vapor pressure (kPa), Δ is slope of the saturation vapor pressure function (kPa °C^-1^), *γ* is psychometric constant (kPa °C^-1^), U_2_ is wind speed at 2 m height (m s^-1^).

### Boosting-based models

Boosting algorithm is a category of the ensemble learning algorithm. The principle of the Boosting algorithm is to first train a weak learner1 from the training set with the initial weight and then update the weight according to the error. When the weight becomes higher, samples with high error rate are more valued in the latter weak learner 2. After adjusting the weight based on the training set, the repetition of single weak learner is performed until the number of weak learners reaches the predetermined number. Finally, the weak learners are integrated through the set strategy (usually by weighted averaging) to obtain the final strong learner for regression or classification purpose [41].

In 1997, Freund proposed the first practical Boosting algorithm-Adaptive Boosting [42], which laid the foundation for Boosting from an idea to a practical approach. Subsequently, Friedman introduced the idea of gradient descent into the Boosting algorithm and then proposed the Gradient Boosting algorithm [35] which is more practical and can handle different loss functions. Based on the above research, Boosting-based model has been continuously developed by researchers and has already been widely used in classification and regression problems. In this study, five Boosting-based models, Adaptive Boosting (ADA), Gradient Boosting Decision Tree(GBDT), Extreme Gradient Boosting(XGB), Light Gradient Boosting Decision Machine(LGB) and Gradient boosting with categorical features support(CAT), are employed to compare their performance of estimating ET_0_ value. All codes of Boosting-based models introduced in this study were written in Python and performed in a laptop with Intel Core i7-9750H CPU @2.60GHz, NVDIA GeForce GTX 1660Ti GPU and 16GB of RAM. For evaluating the performance of each model at the same level of model structure and complexity, only ‘*n_estimators*’ and ‘*learning_rate*’ were set to 500 and 0.05 respectively and other hyper parameters were set to default.

#### Adaptive Boosting (ADA)

The first boosting algorithm, Adaptive Boosting (ADA) was proposed by Freund [42]. AdaBoost assigns equal initial weights to all training data for weak learns training, then updates the weight distribution according to the prediction results. To be more specific, higher weights are assigned to mispredicted samples while lower weights are given to samples predicted correctly, which makes the next training step more focused on mispredicted samples to reduce bias. Above process is repeated until the specified number of iterations or the expected error rate is reached, then all predicted results of the weak learners are added linearly with weights as the final result. The detailed calculation procedures of ADA are described as follows:

For the given dataset $D={\left\{{\text{x}}_{i},{\text{y}}_{i}\right\}}_{i=1}^{M}$, the steps of ADA model for regression problem can be expressed as follows:

(1) Initialize the weight distribution of the training samples as follows:

For *i* = 1,2,3…*M*

$$D_{1}=\left(\omega_{11},\ldots ,\omega_{1i},\ldots ,\omega_{1M}\right),\omega_{1i}=\frac{1}{M}$$ (3)

(2) For *k* (*k* = 1,2,3…*K*), taking *D*_*k*_ as the training set of weak learner *f*_*k*_(*x*) and calculating the following indicators:

(a) Maximum error:

$$E_{k}=\max\left|y_{i}-f_{k}\left(x_{i}\right)\right|,i=1,2,3\ldots M$$ (4)

(b) Relative error of each sample:

$$e_{ki}=\frac{{\left(y_{i}-f_{k}\left(x_{i}\right)\right)}^{2}}{E_{k}}$$ (5)

(c) Regression error rate:

$$e_{k}=\sum_{i=1}^{M}\omega_{ki}e_{ki}$$ (6)

(d) Weight of weak learner *f*_*k*_(*x*):

$$\alpha_{k}=\frac{e_{k}}{1-e_{k}}$$ (7)

(e) Weight distribution of samples is updated as:

$$\omega_{k+1,i}=\frac{\omega_{ki}}{Z_{k}}\alpha_{k}{}^{1-e_{ki}}$$ (8)

Where *Z*_*k*_ is normalizing factor:

$$Z_{k}=\sum_{i=1}^{M}\omega_{ki}\alpha_{k}{}^{1-e_{ki}}$$ (9)

(3) The final strong learner is obtained as:

$$f\left(x\right)=\sum_{m=1}^{M}\left(\text{ln}\left(\frac{1}{\alpha_{m}}\right)\right)g\left(x\right)$$ (10)

Where *g(x)* is the median of all *α*_*m*_*f*_*m*_(*x*), m = 1,2,3…M.

Although ADA is no longer suitable for the current scenario of large sample, high-latitude data usage, its appearance has turned Boosting idea from an initial conjecture into a practical algorithm, which greatly promoted the development of subsequent Boosting-based algorithms.

#### Gradient Boosting Decision Tree (GBDT)

The Gradient Boosting Decision Tree (GBDT) is an iterative decision tree algorithm, proposed by Friedman [35]. The weak learners in GBDT model have strong dependencies between each other and are trained by progressive iterations based on the residuals. The results of all weak learners are added together as the final result, which makes GBDT have great advantages in over-fitting and computational cost fields and also insensitive to data set missing and can reduce bias at the same time. The detailed calculation procedures of GBDT are described as follows:

For the given dataset $D={\left\{{\text{x}}_{i},{\text{y}}_{i}\right\}}_{i=1}^{M}$, the steps of GBDT model for regression problem can be expressed as follows:

(1) Initialize the weak learner:

$$f_{\text{0}}\left(x\right)\text{=arg}\underset{\gamma }{\min}\sum_{i=1}^{M}L\left(y_{i},\gamma \right)$$ (11)

Where L(*y*_*i*_, *γ*) is the loss function.

(2) For *m* (*m* = 1,2,3…*M*) sample in the training set, the residual along the gradient direction is written as:

$$r_{im}\text{=-}{\left[\frac{\partial L\left(y_{i},f\left(x_{i}\right)\right)}{\partial f\left(x_{i}\right)}\right]}_{f\left(x\right)=f_{n-1}\left(x\right)}$$ 12

Where *n* is the number of the estimators (‘*n_estimators*), *n* = 1,2,3…,*N*.

(3) Taking (*x*_*i*_, *r*_*im*_) *i* = 1,2,3…,*m* as the training data of the weak learner n and the leaf node region is *R*_*nj*_, *j* = 1,2,3…,*J*. For this new weak learner, the optimal negative gradient fitting value of each leaf node is calculated as follows:

$$\gamma_{nj}\text{=}\arg\underset{\gamma }{\min}\sum_{x_{i}\in R_{nj}}L\left(y_{i},f_{n-1}\left(x_{i}\right)+\gamma \right)$$ (13)

(4) The model is updated as:

$$f_{n}\left(x\right)=f_{n-1}\left(x\right)+\sum_{j=1}^{J}\gamma_{nj}I\left(x\in R_{nj}\right)$$ (14)

(5) The final strong learner is obtained as:

$$\overset{}{f}\left(x\right)=f_{0}\left(x\right)+\sum_{n=1}^{N}\sum_{j=1}^{J}\gamma_{nj}I\left(x\in R_{nj}\right)$$ (15)

#### Extreme Gradient Boosting (XGB)

Extreme Gradient Boosting (XGB) is an improved algorithm based on GBDT algorithm [36]. Different from the original GBDT model, XGB model obtains the residual by performing second-order Taylor expansion on the cost function, and adds a regularization term to control the complexity of the model at the same time. The addition of regularization terms reduces the variance of the model and makes the model more simplified, making XGB model superior to original GBDT model in terms of weighing the bias-variance tradeoff and preventing overfitting. Also, XGB supports multiple cost functions and parallel operations on feature granularity.

The specific calculation procedures of XGB are described as follows:

(1) Define the objective function as follows:

$$O\text{=}\sum_{i=1}^{n}L\left(y_{i},f\left(x_{i}\right)\right)+\sum_{k=1}^{t}R\left(f_{k}\right)+C$$ (16)

Where *C* is a constant term, which can be commonly omitted and *R*(*f*_*k*_) is the regularization term at the k time iteration, defined as follows:

$$R\left(f_{k}\right)=\alpha H+\frac{1}{2}\eta \sum_{j=1}^{T}\omega_{j}{}^{2}$$ (17)

Where *α* is complexity of leaves, *T* is the number of the leaves, *η* is the penalty parameter and *ωj* is the output result of each leaf node.

(2) Introduce second-order Taylor series of objective function and adopt the mean square error as the loss function, the objective function can be described as follows:

$$O\text{=}\sum_{i=1}^{n}\left[g_{i}\omega_{q\left(x_{i}\right)}+\frac{1}{2}\left(h_{i}\omega_{{}_{q\left(x_{i}\right)}}^{2}\right)\right]+\alpha T+\frac{1}{2}\eta \sum_{j=1}^{T}\omega_{{}_{j}}^{2}$$ (18)

Where $\omega_{q\left(x_{i}\right)}$ is *f*_*k*_, g_*i*_ and h_*i*_ is the first and second derivative of loss function, respectively.

the output result of each leaf node.

(3) Determine the final loss value by summing the loss values of leaf nodes. Therefore, the objective function can be expressed as:

$$O\text{=}\sum_{j=1}^{T}\left[G_{j}\omega_{j}+\frac{1}{2}\left(H_{j}+\eta \right)\omega_{j}^{2}\right]+\alpha T$$ (19)

Where $G_{i}\text{=}\sum_{i\in I_{j}}g_{i}$, $H_{i}\text{=}\sum_{i\in I_{j}}h_{i}$, and *I*_*j*_ indicates all samples in leaf node *j*.

#### Light Gradient Boosting Decision Machine (LGB)

**Fig 1:**
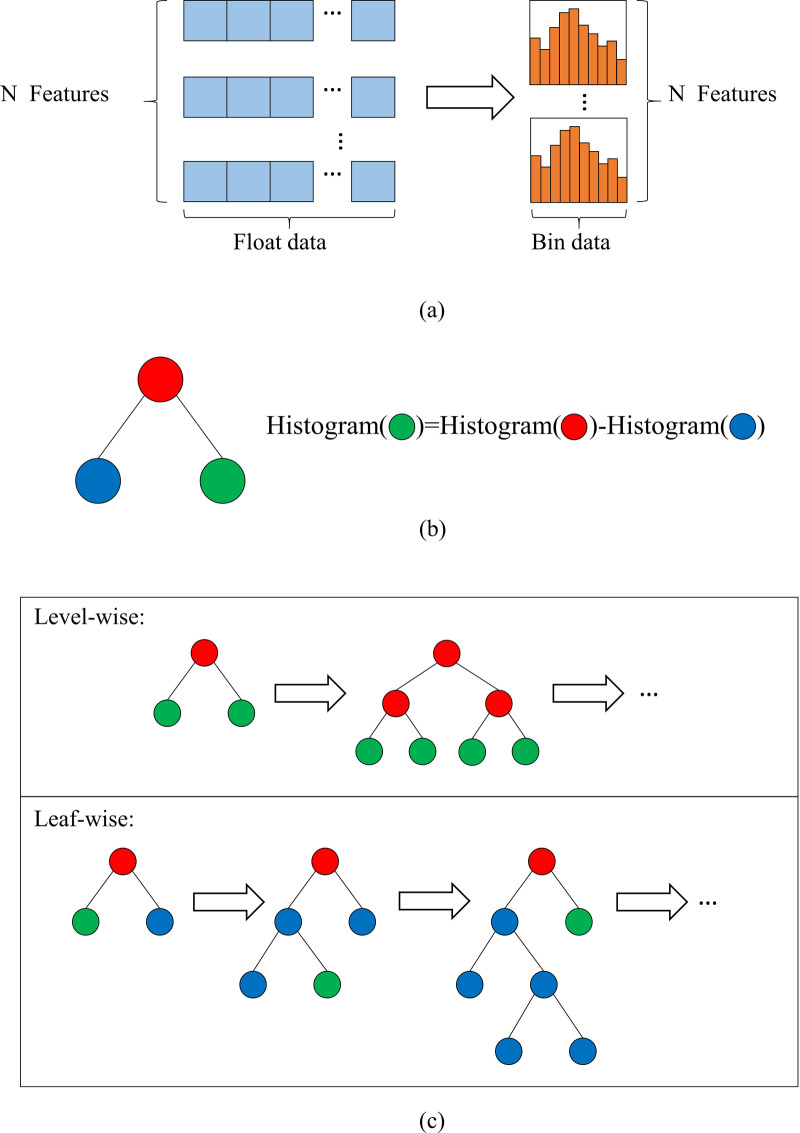
Special process of LGB algorithm. (a) Histogram-based algorithm; (b) Obtain difference value by histogram value; (c) Level-wise and leaf-wise strategies.

Light Gradient Boosting Decision Machine (LGB) is a novel algorithm from Microsoft [43], which has the advantages of lower memory consumption, higher precision and faster training efficiency. Traditional Boosting-based algorithms need to scan all the sample points for each feature to select the best segmentation point, which leads to the model taking too much time in the large sample and high latitude data condition. In order to solve the above problems and further improve the efficiency and scalability of the model, LGB introduces the Gradient-based One-Side Sampling(GOSS) and Exclusive Feature Bundling(EF-B) algorithm. Fig 1 illustrates the special strategy adopted by LGB algorithm and detailed introduction is as follows:

The GOSS algorithm does not use all sample points to calculate the gradient, but instead reserves the sample points with large gradients and performs random sampling on the sample points with small gradients to complete the data sampling in order to maintain the accuracy of information gain. Information gain indicates the expected reduction in entropy caused by splitting the nodes based on attributes, which can be described as follows:

$$IG\left(B,V\right)=En\left(B\right)-\sum_{\upsilon \in Values\left(V\right)}\frac{\left|B_{v}\right|}{B}En\left(B_{v}\right)$$ (20)

$$En\left(B\right)\text{=}\sum_{d=1}^{D}p_{d}\log_{2}p_{d}$$ (21)

Where *En(B)* is the information entropy of the collection *B*, *p*_*d*_ is the ratio of *B* pertaining to category *d*, *D* is the number of categories, *v* is the value of attribute *V* and *B*_*v*_ is the subset of *B* for which attribute has value *v*.

As shown in Fig 1A, the EF-B uses a histogram-based approach, which can discrete floating-point eigenvalues into k integers and construct a histogram of k width. In this way, optimal segmentation point can be found based on the discrete value of histogram with lower memory consumption. In addition, Fig 1B also manifests that the histogram of a single leaf can be obtained by contrasting the histogram of its parent node with that of its sibling node in LGB algorithm, which further increases the speed of the model.

The general process of level-wise and leaf-wise strategies is shown in Fig 1C. Compared with the level-wise strategy, the limited leaf-wise strategy used by LGB could be more effective because it only split at the leaf with the largest information gain and the limited depth can prevent overfitting effectively.

#### Gradient boosting with categorical features support (CAT)

Gradient boosting [44] with categorical features support (CAT) introduces a modified target-based statistics (TBS) to use all data set for training and avoid potential overfitting problem by performing random permutations. To be more specific, CAT first randomly sorts all samples, and then takes a value from a category-based feature. Each sample's feature is converted to a numerical value by taking an average value based on the category label that precedes the sample, and adding priority and weight coefficients of priority. In the process of building new weak learners, CAT first uses the gradient of the sample points before the sample *X*_*n*_ to estimate the model, and then uses these models to calculate the gradient of *X*_*n*_ and update the model. Moreover, CAT uses the oblivious tree as the weak learner, and the index of each leaf node in the oblivious tree can be encoded as a binary vector of length equal to the depth of the tree, which further enhance the model’s ability to resist overfitting.

Compared with XGB and LGB, CAT has following main contributions:

1. Categorical features can be handled automatically by using TBS before training process.
2. Feature dimensions can be enriched by combining the category features according to the relationship between different ones.
3. Overfitting problem can be better resisted by adopting complete oblivious tree.

### Calibration and validation of the models

This study considered limited meteorological variables input combinations as the combination of air temperature data (T_max_ and T_min_) with Rs, RH and U_2_ respectively. In addition, Since extraterrestrial radiation (Ra) is commonly applied to improve the modeling accuracy for estimating ET_0_ with limited input meteorological variables and it is closely related to the geographic data of the station and the number of the days in a year(J), this study also employed J as the input variable to compare with the modeling accuracy improvement brought by Ra and J.

As summarized above, six input meteorological variables combinations were shown in Table 2, these combinations are: (1) T_max_, T_min_, Rs; (2) T_max_, T_min_, RH; (3) T_max_, T_min_, U_2_; (4) T_max_, T_min_; (5) T_max_, T_min_, Ra and (6) T_max_, T_min_, J.

**Table 2:** The input meteorological variables combinations for different models.

Input combination	Input variables	Model abbreviation				
		ADA	GBDT	XGB	LGB	CAT
M1	T_max_, T_min_, Rs	ADA1	GBDT1	XGB1	LGB1	CAT1
M2	T_max_, T_min_, RH	ADA2	GBDT2	XGB2	LGB2	CAT2
M3	T_max_, T_min_, U_2_	ADA3	GBDT3	XGB3	LGB3	CAT3
M4	T_max_, T_min_	ADA4	GBDT4	XGB4	LGB4	CAT4
M5	T_max_, T_min_, Ra	ADA5	GBDT5	XGB5	LGB5	CAT5
M6	T_max_, T_min_, J	ADA6	GBDT6	XGB6	LGB6	CAT6

10-fold cross validation method was used to better evaluate the accuracy of the model and reduce the randomness brought by test samples, and the average value of 10-fold cross-validation result is used as the final performance of the model. In addition, meteorological data from 1997 to 2011, 2012 to 2016 were used as the training and testing set respectively, with a different proportion of training and testing sets from that of 10-fold cross validation stage to analyze model accuracy on daily scale.

### Performance criteria

Present study introduced root mean square error (RMSE), mean absolute error (MAE) and adjusted R^2^ (Adj_R^2^) to evaluate performance of the models [4,24,26,28,45].

$$RMSE=\sqrt{\frac{1}{N}{\sum_{i=1}^{N}\left(ET_{i}^{M}-ET_{i}^{PM}\right)}^{2}}$$ (22)

$$MAE=\frac{1}{N}\sum_{i=1}^{N}\left|ET_{i}^{M}-ET_{i}^{PM}\right|$$ (23)

$$R^{2}=\frac{{\left[\sum_{i=1}^{N}\left(ET_{i}^{PM}-\bar{ET_{0}{}^{PM}}\right)\left(ET_{i}^{M}-\bar{ET_{M}{}^{PM}}\right)\right]}^{2}}{\sum_{i=1}^{N}{\left(ET_{i}^{PM}-\bar{ET_{0}{}^{PM}}\right)}^{2}\sum_{i=1}^{N}{\left(ET_{i}^{M}-\bar{ET_{M}{}^{PM}}\right)}^{2}}$$ (24)

$$Adj\_R^{2}=1-\frac{\left(1-R^{2}\right)\left(N-1\right)}{N-P-1}$$ (25)

Where $ET_{i}^{PM}$ and $ET_{i}^{M}$ are *ET*_0_ values estimated by FAO-56 PM and other models respectively. $\bar{ET_{0}^{PM}}$ and $\bar{ET_{0}^{M}}$ are the mean values of the ET_0_ values estimated by FAO-56 PM model and other models respectively. N and P are the number of test samples and variables, respectively. *i* is the number of *i*-th step, *n* is the number of the total steps. RMSE is in mm day^-1^, with the value range from 0 (optimum value) to +∞ (worst value). MAE is in mm/d, with the value range from 0 (optimum value) to +∞ (worst value). R^2^ and Adj_R^2^ are dimensionless, with the value range from 1 (optimum value) to −∞ (worst value).

## Results

### Comparison of different Boosting-based models with various input combinations on daily scale

The performances of Boosting-based models with daily different meteorological variables inputs at Harbin, Shenyang, Yan 'an, Jinan, Nanjing, Changsha, Chengdu, Kunming, Nanning and Guangzhou stations were illustrated in Tables 3–12, respectively. Tables manifested that the tested models generally had similar performance ranking across 10 stations. For brevity, Harbin, Changsha and Guangzhou were chosen as representatives of TMZ, SMZ and TPMZ respectively to describe in detail.

**Table 3:** Performance of Boosting-based models during 10-fold cross validation and testing stages at Harbin station.

Models	10 Fold cross validation results			Testing results		
	RMSE	MAE	Adj_R^2^	RMSE	MAE	Adj_R^2^
	mm/d	mm/d		mm/d	mm/d	
**T**_**max**_**, T**_**min**_**, Rs**						
ADA1	0.5748	0.4715	0.9019	0.5400	0.4140	0.9042
GBDT1	0.4523	0.2994	0.9400	0.3942	0.2790	0.9489
XGB1	0.4354	0.2937	0.9444	0.3769	0.2784	0.9533
LGB1	0.4335	0.2890	0.9449	0.3721	0.2694	0.9545
CAT1	**0.4288**	**0.2871**	**0.9461**	**0.3699**	**0.2662**	**0.9550**
**T**_**max**_**, T**_**min**_**, RH**						
ADA2	0.6108	0.4857	0.8883	0.6400	0.5263	0.8654
GBDT2	0.4511	0.2994	0.9401	0.4270	0.2843	0.9401
XGB2	0.4411	0.3051	0.9427	0.4228	0.2994	0.9413
LGB2	0.4385	0.2950	0.9433	0.4261	0.2925	0.9403
CAT2	**0.4334**	**0.2919**	**0.9446**	**0.4123**	**0.2844**	**0.9441**
**T**_**max**_**, T**_**min**_**, U**_**2**_						
ADA3	0.7052	0.5417	0.8537	0.6934	0.5441	0.8420
GBDT3	0.6268	0.4417	0.8838	0.6091	0.4275	0.8781
XGB3	0.6108	0.4348	0.8899	0.5955	0.4286	0.8834
LGB3	0.6091	0.4324	0.8906	0.5885	0.4211	0.8862
CAT3	**0.5997**	**0.4259**	**0.8940**	**0.5811**	**0.4132**	**0.8890**
**T**_**max**_**, T**_**min**_						
ADA4	0.7432	0.5585	0.8373	0.7524	0.5557	0.8141
GBDT4	0.6911	0.4821	0.8596	0.6508	0.4656	0.8609
XGB4	0.6817	0.4905	0.8636	0.6488	0.4804	0.8618
LGB4	0.6709	0.4803	0.8684	0.6269	0.4516	0.8709
CAT4	**0.6586**	**0.4657**	**0.8726**	**0.6228**	**0.4466**	**0.8726**
**T**_**max**_**, T**_**min**_**, Ra**						
ADA5	0.6812	0.5107	0.8632	0.6806	0.5286	0.8478
GBDT5	0.5581	0.3706	0.9086	0.5358	0.3668	0.9057
XGB5	0.5330	0.3584	0.9168	0.5094	0.3563	0.9147
LGB5	0.5291	0.3543	0.9181	0.5019	0.3460	0.9172
CAT5	**0.5224**	**0.3507**	**0.9201**	**0.4957**	**0.3385**	**0.9193**
**T**_**max**_**, T**_**min**_**, J**						
ADA6	0.6429	0.4783	0.8782	0.6426	0.4856	0.8643
GBDT6	0.5394	0.3600	0.9147	0.5185	0.3536	0.9117
XGB6	0.5235	0.3539	0.9197	0.5075	0.3554	0.9153
LGB6	0.5228	0.3516	0.9199	0.4928	0.3424	0.9202
CAT6	**0.5122**	**0.3473**	**0.9231**	**0.4897**	**0.3395**	**0.9212**

**Table 4:** Performance of Boosting-based models during 10-fold cross validation and testing stages at Shenyang station.

Models	10 Fold cross validation results			Testing results		
	RMSE	MAE	Adj_R^2^	RMSE	MAE	Adj_R^2^
	mm/d	mm/d		mm/d	mm/d	
**T**_**max**_**, T**_**min**_**, Rs**						
ADA1	0.6376	0.5208	0.8792	0.6089	0.4706	0.8795
GBDT1	0.5315	0.3657	0.9184	0.4912	0.3413	0.9216
XGB1	0.5173	0.3630	0.9225	0.4785	0.3399	0.9256
LGB1	0.5139	0.3567	0.9236	0.4721	0.3307	0.9276
CAT1	**0.5020**	**0.3506**	**0.9272**	**0.4656**	**0.3255**	**0.9295**
**T**_**max**_**, T**_**min**_**, RH**						
ADA2	0.6737	0.5374	0.8635	0.6533	0.5281	0.8613
GBDT2	0.5224	0.3571	0.9202	0.4875	0.3350	0.9228
XGB2	0.5085	0.3603	0.9242	0.4813	0.3417	0.9247
LGB2	0.5071	0.3528	0.9246	0.4801	0.3309	0.9251
CAT2	**0.5027**	**0.3485**	**0.9259**	**0.4763**	**0.3311**	**0.9264**
**T**_**max**_**, T**_**min**_**, U**_**2**_						
ADA3	0.7903	0.6300	0.8157	0.7637	0.6239	0.8104
GBDT3	0.6644	0.4896	0.8695	0.6189	0.4626	0.8755
XGB3	0.6488	0.4820	0.8758	0.5992	0.4545	0.8833
LGB3	0.6477	0.4797	0.8763	0.5933	0.4481	0.8856
CAT3	**0.6334**	**0.4711**	**0.8814**	**0.5894**	**0.4491**	**0.8871**
**T**_**max**_**, T**_**min**_						
ADA4	0.8371	0.6621	0.7935	0.8270	0.6298	0.7779
GBDT4	0.7469	0.5449	0.8375	0.7002	0.5226	0.8408
XGB4	0.7419	0.5578	0.8391	0.6952	0.5368	0.8430
LGB4	0.7218	0.5371	0.8485	0.6633	0.5031	0.8571
CAT4	**0.7087**	**0.5263**	**0.8532**	**0.6584**	**0.4994**	**0.8592**
**T**_**max**_**, T**_**min**_**, Ra**						
ADA5	0.7546	0.5880	0.8322	0.7240	0.5741	0.8297
GBDT5	0.6150	0.4283	0.8924	0.5693	0.4098	0.8947
XGB5	0.5879	0.4166	0.8992	0.5506	0.4017	0.9015
LGB5	0.5835	0.4122	0.9009	0.5460	0.3945	0.9031
CAT5	**0.5731**	**0.4073**	**0.9043**	**0.5446**	**0.3928**	**0.9036**
**T**_**max**_**, T**_**min**_**, J**						
ADA6	0.7419	0.5807	0.8381	0.7088	0.5623	0.8367
GBDT6	0.5970	0.4191	0.8959	0.5626	0.4021	0.8971
XGB6	0.5794	0.4130	0.9019	0.5462	0.3987	0.9030
LGB6	0.5752	0.4087	0.9034	0.5439	0.3921	0.9039
CAT6	**0.5684**	**0.4068**	**0.9057**	**0.5385**	**0.3921**	**0.9058**

**Table 5:** Performance of Boosting-based models during 10-fold cross validation and testing stages at Yan’an station.

Models	10 Fold cross validation results			Testing results		
	RMSE	MAE	Adj_R^2^	RMSE	MAE	Adj_R^2^
	mm/d	mm/d		mm/d	mm/d	
**T**_**max**_**, T**_**min**_**, Rs**						
ADA1	0.6368	0.5079	0.8905	0.6544	0.5167	0.8700
GBDT1	0.5508	0.4074	0.9180	0.5198	0.3963	0.9180
XGB1	0.5338	0.3975	0.9230	0.4962	0.3842	0.9252
LGB1	0.5299	0.3930	0.9241	0.4876	0.3777	0.9278
CAT1	**0.5251**	**0.3895**	**0.9254**	**0.4829**	**0.3732**	**0.9292**
**T**_**max**_**, T**_**min**_**, RH**						
ADA2	0.6503	0.5274	0.8851	0.6819	0.5633	0.8588
GBDT2	0.5592	0.3997	0.9156	0.5238	0.3809	0.9167
XGB2	0.5361	0.3935	0.9225	0.5126	0.3826	0.9202
LGB2	0.5377	0.3893	0.9219	0.5166	0.3804	0.9190
CAT2	**0.5280**	**0.3830**	**0.9248**	**0.5016**	**0.3679**	**0.9236**
**T**_**max**_**, T**_**min**_**, U**_**2**_						
ADA3	0.8212	0.6553	0.8172	0.8121	0.6559	0.7998
GBDT3	0.6703	0.5010	0.8785	0.6402	0.4793	0.8756
XGB3	0.6560	0.4919	0.8836	0.6262	0.4714	0.8810
LGB3	0.6524	0.4890	0.8849	0.6211	0.4655	0.8829
CAT3	**0.6411**	**0.4817**	**0.8888**	**0.6117**	**0.4597**	**0.8864**
**T**_**max**_**, T**_**min**_						
ADA4	0.8948	0.7024	0.7832	0.8925	0.6975	0.7583
GBDT4	0.8265	0.6165	0.8160	0.7883	0.5962	0.8115
XGB4	0.8096	0.6191	0.8232	0.7723	0.6023	0.8190
LGB4	0.8013	0.6078	0.8275	0.7480	0.5736	0.8302
CAT4	**0.7890**	**0.5960**	**0.8323**	**0.7372**	**0.5642**	**0.8351**
**T**_**max**_**, T**_**min**_**, Ra**						
ADA5	0.7998	0.6248	0.8269	0.7930	0.6194	0.8091
GBDT5	0.6810	0.4994	0.8756	0.6599	0.4852	0.8678
XGB5	0.6614	0.4888	0.8819	0.6354	0.4720	0.8774
LGB5	0.6598	0.4878	0.8825	0.6275	0.4668	0.8804
CAT5	**0.6499**	**0.4818**	**0.8860**	**0.6157**	**0.4566**	**0.8849**
**T**_**max**_**, T**_**min**_**, J**						
ADA6	0.7835	0.6064	0.8340	0.7825	0.6067	0.8141
GBDT6	0.6742	0.4964	0.8774	0.6494	0.4812	0.8720
XGB6	0.6550	0.4870	0.8842	0.6326	0.4720	0.8785
LGB6	0.6523	0.4844	0.8852	0.6209	0.4633	0.8830
CAT6	**0.6397**	**0.4755**	**0.8896**	**0.6118**	**0.4570**	**0.8864**

**Table 6:** Performance of Boosting-based models during 10-fold cross validation and testing stages at Ji’nan station.

Models	10 Fold cross validation results			Testing results		
	RMSE	MAE	Adj_R^2^	RMSE	MAE	Adj_R^2^
	mm/d	mm/d		mm/d	mm/d	
**T**_**max**_**, T**_**min**_**, Rs**						
ADA1	0.7516	0.6139	0.8547	0.6986	0.5436	0.8637
GBDT1	0.6115	0.4357	0.9047	0.5679	0.4050	0.9100
XGB1	0.5977	0.4309	0.9089	0.5423	0.3940	0.9179
LGB1	0.5924	0.4249	0.9106	0.5359	0.3870	0.9198
CAT1	**0.5861**	**0.4198**	**0.9124**	**0.5259**	**0.3789**	**0.9228**
**T**_**max**_**, T**_**min**_**, RH**						
ADA2	0.8077	0.6538	0.8315	0.7889	0.6382	0.8262
GBDT2	0.6413	0.4586	0.8952	0.6132	0.4384	0.8950
XGB2	0.6143	0.4513	0.9034	0.5860	0.4360	0.9041
LGB2	0.6124	0.4444	0.9042	0.5769	0.4209	0.9071
CAT2	**0.6044**	**0.4388**	**0.9066**	**0.5766**	**0.4204**	**0.9072**
**T**_**max**_**, T**_**min**_**, U**_**2**_						
ADA3	0.8731	0.6815	0.8043	0.8215	0.6502	0.8115
GBDT3	0.7067	0.5301	0.8717	0.6672	0.5015	0.8757
XGB3	0.6931	0.5226	0.8767	0.6472	0.4891	0.8830
LGB3	0.6907	0.5206	0.8776	0.6454	0.4877	0.8837
CAT3	**0.6811**	**0.5126**	**0.8810**	**0.6334**	**0.4755**	**0.8880**
**T**_**max**_**, T**_**min**_						
ADA4	0.9552	0.7496	0.7658	0.9106	0.7133	0.7686
GBDT4	0.8563	0.6407	0.8135	0.8108	0.6155	0.8166
XGB4	0.8500	0.6508	0.8159	0.7829	0.6107	0.8289
LGB4	0.8330	0.6297	0.8240	0.7651	0.5826	0.8366
CAT4	**0.8204**	**0.6183**	**0.8287**	**0.7471**	**0.5705**	**0.8442**
**T**_**max**_**, T**_**min**_**, Ra**						
ADA5	0.8630	0.6652	0.8089	0.8226	0.6402	0.8111
GBDT5	0.7330	0.5349	0.8626	0.6907	0.5090	0.8668
XGB5	0.7139	0.5236	0.8698	0.6723	0.4996	0.8738
LGB5	0.7099	0.5200	0.8713	0.6629	0.4916	0.8773
CAT5	**0.7042**	**0.5146**	**0.8734**	**0.6520**	**0.4847**	**0.8813**
**T**_**max**_**, T**_**min**_**, J**						
ADA6	0.8357	0.6445	0.8215	0.7851	0.6114	0.8279
GBDT6	0.7259	0.5306	0.8654	0.6957	0.5156	0.8649
XGB6	0.7044	0.5182	0.8732	0.6643	0.4957	0.8768
LGB6	0.6978	0.5126	0.8757	0.6569	0.4898	0.8795
CAT6	**0.6877**	**0.5060**	**0.8791**	**0.6479**	**0.4801**	**0.8828**

**Table 7:** Performance of Boosting-based models during 10-fold cross validation and testing stages at Nanjing station.

Models	10 Fold cross validation results			Testing results		
	RMSE	MAE	Adj_R^2^	RMSE	MAE	Adj_R^2^
	mm/d	mm/d		mm/d	mm/d	
**T**_***max***_**, *T***_***min***_**, *Rs***						
ADA1	0.5564	0.4663	0.8590	0.5494	0.4638	0.8597
GBDT1	0.3918	0.2850	0.9315	0.3605	0.2721	0.9396
XGB1	0.3832	0.2816	0.9345	0.3505	0.2658	0.9429
LGB1	0.3798	0.2784	0.9356	0.3476	0.2630	0.9438
CAT1	**0.3740**	**0.2743**	**0.9376**	**0.3406**	**0.2593**	**0.9461**
**T**_***max***_**, *T***_***min***_**, *RH***						
ADA2	0.6397	0.5326	0.8157	0.5993	0.4761	0.8330
GBDT2	0.5462	0.3969	0.8665	0.5175	0.3749	0.8755
XGB2	0.5269	0.3923	0.8757	0.4994	0.3762	0.8840
LGB2	0.5264	0.3880	0.8760	0.4970	0.3659	0.8851
CAT2	**0.5194**	**0.3830**	**0.8793**	**0.4917**	**0.3602**	**0.8876**
**T**_***max***_**, *T***_***min***_**, *U***_***2***_						
ADA3	0.7774	0.6195	0.7265	0.7634	0.6113	0.7290
GBDT3	0.6851	0.5174	0.7893	0.6453	0.4913	0.8064
XGB3	0.6647	0.5042	0.8016	0.6226	0.4792	0.8198
LGB3	0.6613	0.5021	0.8038	0.6130	0.4722	0.8252
CAT3	**0.6548**	**0.4956**	**0.8076**	**0.6065**	**0.4642**	**0.8290**
**T**_***max***_**, *T***_***min***_						
ADA4	0.9803	0.7305	0.5812	0.7509	0.6003	0.7380
GBDT4	0.7695	0.5441	0.7446	0.6737	0.5172	0.7891
XGB4	0.6927	0.5365	0.7849	0.6583	0.5171	0.7986
LGB4	0.6785	0.5170	0.7940	0.6359	0.4901	0.8121
CAT4	**0.6699**	**0.5081**	**0.7992**	**0.6274**	**0.4820**	**0.8171**
**T**_***max***_**, *T***_***min***_**, *Ra***						
ADA5	0.7482	0.5925	0.7474	0.7352	0.5801	0.7487
GBDT5	0.6311	0.4661	0.8215	0.6141	0.4575	0.8247
XGB5	0.6106	0.4534	0.8328	0.5935	0.4470	0.8362
LGB5	0.6062	0.4501	0.8352	0.5875	0.4431	0.8395
CAT5	**0.6014**	**0.4453**	**0.8379**	**0.5738**	**0.4298**	**0.8469**
**T**_***max***_**, *T***_***min***_**, *J***						
ADA6	0.7645	0.6077	0.7359	0.7557	0.6005	0.7345
GBDT6	0.6243	0.4609	0.8253	0.6072	0.4536	0.8286
XGB6	0.6030	0.4479	0.8370	0.5850	0.4433	0.8408
LGB6	0.5980	0.4454	0.8397	0.5781	0.4387	0.8446
CAT6	**0.5914**	**0.4380**	**0.8431**	**0.5665**	**0.4244**	**0.8508**

**Table 8:** Performance of Boosting-based models during 10-fold cross validation and testing stages at Changsha station.

Models	10 Fold cross validation results			Testing results		
	RMSE	MAE	Adj_R^2^	RMSE	MAE	Adj_R^2^
	mm/d	mm/d		mm/d	mm/d	
**T**_***max***_**, *T***_***min***_**, *Rs***						
ADA1	0.4275	0.3527	0.8976	0.4433	0.3658	0.8894
GBDT1	0.2865	0.2037	0.9536	0.2715	0.1989	0.9585
XGB1	0.2790	0.2011	0.9559	0.2641	0.1952	0.9607
LGB1	0.2790	0.1994	0.9559	0.2654	0.1935	0.9604
CAT1	**0.2746**	**0.1959**	**0.9573**	**0.2569**	**0.1881**	**0.9628**
**T**_***max***_**, *T***_***min***_**, *RH***						
ADA2	0.6134	0.4873	0.7908	0.5861	0.4568	0.8066
GBDT2	0.5744	0.4212	0.8161	0.5355	0.3968	0.8386
XGB2	0.5528	0.4121	0.8298	0.5141	0.3879	0.8512
LGB2	0.5531	0.4098	0.8296	0.5086	0.3805	0.8544
CAT2	**0.5462**	**0.4040**	**0.8338**	**0.5021**	**0.3731**	**0.8581**
**T**_***max***_**, *T***_***min***_**, *U***_***2***_						
ADA3	0.7151	0.5802	0.7143	0.7130	0.5798	0.7138
GBDT3	0.6466	0.4789	0.7667	0.6281	0.4700	0.7779
XGB3	0.6258	0.4693	0.7816	0.6041	0.4586	0.7945
LGB3	0.6257	0.4684	0.7818	0.6018	0.4553	0.7961
CAT3	**0.6176**	**0.4623**	**0.7873**	**0.5978**	**0.4480**	**0.7988**
**T**_***max***_**, *T***_***min***_						
ADA4	0.7174	0.5808	0.7129	0.6982	0.5617	0.7257
GBDT4	0.6622	0.4968	0.7558	0.6394	0.4870	0.7700
XGB4	0.6484	0.5009	0.7659	0.6319	0.4954	0.7754
LGB4	0.6421	0.4913	0.7713	0.6197	0.4753	0.7840
CAT4	**0.6335**	**0.4813**	**0.7767**	**0.6117**	**0.4672**	**0.7895**
**T**_***max***_**, *T***_***min***_**, *Ra***						
ADA5	0.7158	0.5807	0.7138	0.7172	0.5841	0.7104
GBDT5	0.6276	0.4606	0.7802	0.6169	0.4533	0.7858
XGB5	0.6053	0.4487	0.7956	0.5938	0.4444	0.8015
LGB5	0.6035	0.4477	0.7969	0.5912	0.4407	0.8032
CAT5	**0.5943**	**0.4394**	**0.8030**	**0.5834**	**0.4321**	**0.8084**
**T**_***max***_**, *T***_***min***_**, *J***						
ADA6	0.7163	0.5798	0.7133	0.7133	0.5782	0.7136
GBDT6	0.6188	0.4540	0.7863	0.6081	0.4530	0.7918
XGB6	0.5978	0.4430	0.8007	0.5896	0.4437	0.8043
LGB6	0.5962	0.4415	0.8018	0.5870	0.4392	0.8061
CAT6	**0.5870**	**0.4324**	**0.8079**	**0.5754**	**0.4271**	**0.8136**

**Table 9:** Performance of Boosting-based models during 10-fold cross validation and testing stages at Chengdu station.

Models	10 Fold cross validation results			Testing results		
	RMSE	MAE	Adj_R^2^	RMSE	MAE	Adj_R^2^
	mm/d	mm/d		mm/d	mm/d	
**T**_**max**_**, T**_**min**_**, Rs**						
ADA1	0.5473	0.4533	0.8515	0.5506	0.4556	0.8458
GBDT1	0.3697	0.2630	0.9323	0.3640	0.2583	0.9326
XGB1	0.3595	0.2585	0.9360	0.3543	0.2528	0.9361
LGB1	0.3582	0.2556	0.9364	0.3516	0.2508	0.9371
CAT1	**0.3521**	**0.2499**	**0.9385**	**0.3413**	**0.2439**	**0.9407**
**T**_**max**_**, T**_**min**_**, RH**						
ADA2	0.5962	0.4731	0.8247	0.5907	0.4662	0.8225
GBDT2	0.5552	0.4074	0.8476	0.5501	0.4005	0.8461
XGB2	0.5425	0.4042	0.8545	0.5201	0.3891	0.8624
LGB2	0.5370	0.3978	0.8574	0.5235	0.3852	0.8606
CAT2	**0.5337**	**0.3943**	**0.8592**	**0.5109**	**0.3746**	**0.8672**
**T**_**max**_**, T**_**min**_**, U**_**2**_						
ADA3	0.6889	0.5560	0.7665	0.6831	0.5553	0.7626
GBDT3	0.6100	0.4645	0.8166	0.6092	0.4681	0.8112
XGB3	0.5914	0.4548	0.8277	0.5908	0.4595	0.8224
LGB3	0.5898	0.4529	0.8286	0.5881	0.4576	0.8240
CAT3	**0.5835**	**0.4483**	**0.8323**	**0.5753**	**0.4455**	**0.8316**
**T**_**max**_**, T**_**min**_						
ADA4	0.7012	0.5602	0.7582	0.7124	0.5639	0.7419
GBDT4	0.6482	0.4973	0.7933	0.6495	0.5004	0.7855
XGB4	0.6369	0.4984	0.8006	0.6376	0.5016	0.7933
LGB4	0.6288	0.4910	0.8060	0.6330	0.4933	0.7963
CAT4	**0.6197**	**0.4808**	**0.8110**	**0.6206**	**0.4820**	**0.8042**
**T**_**max**_**, T**_**min**_**, Ra**						
ADA5	0.6824	0.5470	0.7703	0.6857	0.5549	0.7608
GBDT5	0.6159	0.4711	0.8125	0.6267	0.4753	0.8002
XGB5	0.5944	0.4593	0.8256	0.5967	0.4606	0.8189
LGB5	0.5948	0.4588	0.8253	0.5953	0.4587	0.8197
CAT5	**0.5869**	**0.4521**	**0.8300**	**0.5795**	**0.4474**	**0.8292**
**T**_**max**_**, T**_**min**_**, J**						
ADA6	0.6739	0.5376	0.7760	0.6697	0.5414	0.7719
GBDT6	0.6135	0.4689	0.8141	0.6180	0.4750	0.8057
XGB6	0.5963	0.4599	0.8247	0.5943	0.4594	0.8203
LGB6	0.5949	0.4590	0.8255	0.5916	0.4574	0.8219
CAT6	**0.5881**	**0.4531**	**0.8294**	**0.5800**	**0.4500**	**0.8288**

**Table 10:** Performance of Boosting-based models during 10-fold cross validation and testing stages at Kunming station.

Models	10 Fold cross validation results			Testing results		
	RMSE	MAE	Adj_R^2^	RMSE	MAE	Adj_R^2^
	mm/d	mm/d		mm/d	mm/d	
**T**_**max**_**, T**_**min**_**, Rs**						
ADA1	0.4682	0.3851	0.8682	0.4675	0.3742	0.8841
GBDT1	0.3507	0.2514	0.9264	0.3735	0.2696	0.9260
XGB1	0.3415	0.2469	0.9301	0.3648	0.2624	0.9294
LGB1	0.3408	0.2457	0.9303	0.3655	0.2616	0.9291
CAT1	**0.3338**	**0.2417**	**0.9331**	**0.3557**	**0.2538**	**0.9329**
**T**_**max**_**, T**_**min**_**, RH**						
ADA2	0.4430	0.3525	0.8816	0.5061	0.3937	0.8642
GBDT2	0.4156	0.3253	0.8962	0.4346	0.3354	0.8998
XGB2	0.4039	0.3168	0.9020	0.4199	0.3262	0.9065
LGB2	0.4028	0.3158	0.9025	0.4166	0.3236	0.9079
CAT2	**0.3977**	**0.3106**	**0.9049**	**0.4110**	**0.3173**	**0.9104**
**T**_**max**_**, T**_**min**_**, U**_**2**_						
ADA3	0.5864	0.4690	0.7933	0.6212	0.4958	0.7953
GBDT3	0.4982	0.3868	0.8503	0.5096	0.4015	0.8622
XGB3	0.4887	0.3807	0.8559	0.4982	0.3925	0.8683
LGB3	0.4871	0.3791	0.8569	0.4998	0.3941	0.8675
CAT3	**0.4785**	**0.3721**	**0.8618**	**0.4890**	**0.3819**	**0.8732**
**T**_**max**_**, T**_**min**_						
ADA4	0.6290	0.5001	0.7630	0.6779	0.5351	0.7564
GBDT4	0.5791	0.4494	0.7988	0.5890	0.4603	0.8161
XGB4	0.5665	0.4437	0.8076	0.5785	0.4570	0.8226
LGB4	0.5633	0.4398	0.8103	0.5720	0.4476	0.8266
CAT4	**0.5542**	**0.4314**	**0.8157**	**0.5620**	**0.4387**	**0.8325**
**T**_**max**_**, T**_**min**_**, Ra**						
ADA5	0.6152	0.4864	0.7731	0.6373	0.5038	0.7845
GBDT5	0.5087	0.3899	0.8448	0.5250	0.4059	0.8538
XGB5	0.5007	0.3828	0.8498	0.5118	0.3947	0.8611
LGB5	0.4996	0.3829	0.8504	0.5162	0.3982	0.8587
CAT5	**0.4914**	**0.3766**	**0.8551**	**0.4906**	**0.3768**	**0.8724**
**T**_**max**_**, T**_**min**_**, J**						
ADA6	0.5392	0.4227	0.8254	0.5712	0.4467	0.8269
GBDT6	0.4735	0.3662	0.8654	0.4933	0.3831	0.8709
XGB6	0.4607	0.3561	0.8726	0.4772	0.3716	0.8792
LGB6	0.4602	0.3558	0.8729	0.4772	0.3719	0.8792
CAT6	**0.4507**	**0.3486**	**0.8780**	**0.4624**	**0.3592**	**0.8866**

**Table 11:** Performance of Boosting-based models during 10-fold cross validation and testing stages at Nanning station.

Models	10 Fold cross validation results			Testing results		
	RMSE	MAE	Adj_R^2^	RMSE	MAE	Adj_R^2^
	mm/d	mm/d		mm/d	mm/d	
**T**_**max**_**, T**_**min**_**, Rs**						
ADA1	0.5397	0.4489	0.7728	0.5938	0.5139	0.7500
GBDT1	0.4300	0.3023	0.8540	0.4247	0.3110	0.8721
XGB1	0.4152	0.2952	0.8638	0.4043	0.3040	0.8841
LGB1	0.4149	0.2935	0.8640	0.4016	0.3003	0.8857
CAT1	**0.4105**	**0.2916**	**0.8667**	**0.3932**	**0.2942**	**0.8904**
**T**_**max**_**, T**_**min**_**, RH**						
ADA2	0.5919	0.4837	0.7254	0.6139	0.4964	0.7327
GBDT2	0.5260	0.4050	0.7812	0.4995	0.3778	0.8231
XGB2	0.5169	0.4029	0.7893	0.4900	0.3797	0.8298
LGB2	0.5104	0.3946	0.7942	0.4796	0.3649	0.8369
CAT2	**0.5060**	**0.3902**	**0.7978**	**0.4768**	**0.3638**	**0.8388**
**T**_**max**_**, T**_**min**_**, U**_**2**_						
ADA3	0.7424	0.6064	0.5696	0.7750	0.6370	0.5742
GBDT3	0.6539	0.5113	0.6642	0.6455	0.5088	0.7045
XGB3	0.6412	0.5036	0.6774	0.6376	0.5039	0.7118
LGB3	0.6399	0.5023	0.6788	0.6377	0.5050	0.7116
CAT3	**0.6344**	**0.4972**	**0.6842**	**0.6323**	**0.4984**	**0.7165**
**T**_**max**_**, T**_**min**_						
ADA4	0.7500	0.6074	0.5613	0.7671	0.6218	0.5830
GBDT4	0.7145	0.5614	0.5995	0.7165	0.5661	0.6362
XGB4	0.6923	0.5490	0.6247	0.6903	0.5587	0.6623
LGB4	0.6903	0.5470	0.6280	0.6812	0.5478	0.6712
CAT4	**0.6847**	**0.5417**	**0.6327**	**0.6747**	**0.5444**	**0.6774**
**T**_**max**_**, T**_**min**_**, Ra**						
ADA5	0.7374	0.5984	0.5752	0.7689	0.6298	0.5808
GBDT5	0.6694	0.5241	0.6466	0.6677	0.5308	0.6839
XGB5	0.6538	0.5146	0.6635	0.6482	0.5221	0.7021
LGB5	0.6553	0.5168	0.6622	0.6472	0.5217	0.7030
CAT5	**0.6486**	**0.5108**	**0.6686**	**0.6401**	**0.5164**	**0.7095**
**T**_**max**_**, T**_**min**_**, J**						
ADA6	0.7472	0.6066	0.5638	0.7834	0.6411	0.5648
GBDT6	0.6390	0.4957	0.6792	0.6405	0.5047	0.7092
XGB6	0.6236	0.4849	0.6948	0.6221	0.4959	0.7256
LGB6	0.6245	0.4863	0.6941	0.6222	0.4949	0.7255
CAT6	**0.6165**	**0.4810**	**0.7011**	**0.6062**	**0.4833**	**0.7395**

**Table 12:** Performance of Boosting-based models during 10-fold cross validation and testing stages at Guangzhou station.

Models	10 Fold cross validation results			Testing results		
	RMSE	MAE	Adj_R^2^	RMSE	MAE	Adj_R^2^
	mm/d	mm/d		mm/d	mm/d	
**T**_**max**_**, T**_**min**_**, Rs**						
ADA1	0.4997	0.4115	0.7624	0.5269	0.4457	0.7515
GBDT1	0.3890	0.2610	0.8539	0.3751	0.2614	0.8740
XGB1	0.3778	0.2577	0.8618	0.3620	0.2613	0.8827
LGB1	0.3760	0.2557	0.8629	0.3585	0.2562	0.8849
CAT1	**0.3708**	**0.2516**	**0.8664**	**0.3528**	**0.2533**	**0.8886**
**T**_**max**_**, T**_**min**_**, RH**						
ADA2	0.6114	0.5029	0.6453	0.5971	0.4804	0.6808
GBDT2	0.5396	0.4172	0.7228	0.5243	0.3938	0.7539
XGB2	0.5334	0.4186	0.7292	0.5013	0.3846	0.7750
LGB2	0.5253	0.4068	0.7374	0.5005	0.3783	0.7757
CAT2	**0.5193**	**0.4036**	**0.7432**	**0.4891**	**0.3697**	**0.7858**
**T**_**max**_**, T**_**min**_**, U**_**2**_						
ADA3	0.7658	0.6215	0.4457	0.7854	0.6315	0.4478
GBDT3	0.6596	0.5088	0.5866	0.6558	0.5006	0.6149
XGB3	0.6422	0.4990	0.6086	0.6341	0.4875	0.6400
LGB3	0.6407	0.4988	0.6106	0.6296	0.4853	0.6451
CAT3	**0.6322**	**0.4906**	**0.6207**	**0.6167**	**0.4760**	**0.6596**
**T**_**max**_**, T**_**min**_						
ADA4	0.7593	0.6152	0.4558	0.7797	0.6181	0.4561
GBDT4	0.7109	0.5574	0.5210	0.6976	0.5467	0.5645
XGB4	0.6944	0.5501	0.5439	0.6736	0.5365	0.5940
LGB4	0.6872	0.5422	0.5542	0.6654	0.5255	0.6038
CAT4	**0.6794**	**0.5336**	**0.5625**	**0.6555**	**0.5169**	**0.6156**
**T**_**max**_**, T**_**min**_**, Ra**						
ADA5	0.7622	0.6171	0.4512	0.7871	0.6389	0.4453
GBDT5	0.6737	0.5221	0.5688	0.6547	0.5084	0.6162
XGB5	0.6592	0.5125	0.5874	0.6427	0.5037	0.6302
LGB5	0.6566	0.5110	0.5904	0.6365	0.4990	0.6373
CAT5	**0.6494**	**0.5036**	**0.5992**	**0.6249**	**0.4879**	**0.6504**
**T**_**max**_**, T**_**min**_**, J**						
ADA6	0.7580	0.6138	0.4574	0.7875	0.6386	0.4449
GBDT6	0.6450	0.4946	0.6047	0.6294	0.4838	0.6453
XGB6	0.6312	0.4865	0.6217	0.6127	0.4760	0.6639
LGB6	0.6282	0.4843	0.6252	0.6083	0.4738	0.6687
CAT6	**0.6171**	**0.4745**	**0.6382**	**0.5909**	**0.4560**	**0.6874**

As shown in Table 3, CAT models generally achieved the best performance (on average RMSE of 0.5259 mm d^-1^, MAE of 0.3614 mm d^-1^ and Adj_R^2^ of 0.9168) among all the tested models with all input combinations at Harbin station (TMZ), followed by LGB (on average RMSE of 0.5430 mm d^-1^, MAE of 0.3671 mm d^-1^ and Adj_R^2^ of 0.9142) and XGB (on average RMSE of 0.5376 mm d^-1^, MAE of 0.3727 mm d^-1^ and Adj_R^2^ of 0.9128). The GBDT models could also achieve acceptable precision (on average RMSE of 0.5618 mm d^-1^, MAE of 0.3883 mm d^-1^ and Adj_R^2^ of 0.9041), while the original ADA models had the relatively worst performance (on average RMSE of 0.6597 mm d^-1^, MAE of 0.5077 mm d^-1^ and Adj_R^2^ of 0.8704).

During the 10-fold cross validation stage, models with M1(Rs) input (with RMSE ranged from 0.4288–0.5748 mm d^-1^, MAE ranged from 0.2871–0.4715 mm d^-1^, Adj_R^2^ ranged from 0.9019–0.9461) and M2(RH) input (with RMSE ranged from 0.4334–0.6108 mm d^-1^, MAE ranged from 0.2919–0.4857 mm d^-1^, Adj_R^2^ ranged from 0.8883–0.9446) performed the best. When only T_max_ and T_min_ data were inputted, models based on M4 input achieved the worst precision (with RMSE ranged from 0.4288–0.5748 mm d^-1^, MAE ranged from 0.2871–0.4715 mm d^-1^, Adj_R^2^ ranged from 0.8373–0.8726), followed by models based on M3(U_2_) input (with RMSE ranged from 0.5997–0.7052 mm d^-1^, MAE ranged from 0.4259–0.5417 mm d^-1^, Adj_R^2^ ranged from 0.8537–0.8940). It is worth to see that models based on M5(Ra) and M6(J), which are combinations of temperature data with Ra and J respectively, could achieve better performance than models based on M4 and even models based on M3 input. In addition, models based on M6 (with RMSE ranged from 0.5122–0.6429 mm d^-1^, MAE ranged from 0.3473–0.4783 mm d^-1^, Adj_R^2^ ranged from 0. 8782–0.9231) could obtain slightly better accuracy than models based on M5 (with RMSE ranged from 0.5224–0.6812 mm d^-1^, MAE ranged from 0.3507–0.5107 mm d^-1^, Adj_R^2^ ranged from 0.8632–0.9201).

The performance of tested models at Changsha station (SMZ) was demonstrated in Table 6. The accuracy ranking of various Boosting-based models was same as that of Harbin station, which was in the order of CAT, LGB, XGB, GBDT and ADA. However, the overall simulation accuracy suffered a slight decrease compared with the performance of models at Harbin station, the average Adj_R^2^ value of ADA, GBDT, XGB, LGB and CAT decreased by 13.02%, 11.22%, 10.00%, 10.00% and 9.72% respectively. Particularly, models based on M1 combination achieved the best precision (with RMSE ranged from 0.2746–0.4275 mm d^-1^, MAE ranged from 0.1959–0.3527 mm d^-1^, Adj_R^2^ ranged from 0.8976–0.9573),which was far ahead of models with other input combinations. The above results could also be found in the testing stage.

Table 12 showed the performance of tested models at Guangzhou station (TPMZ). Same performance ranking of tested models could also be found at Guangzhou station, but overall simulation accuracy suffered a more significant decrease, the average Adj_R^2^ value of ADA, GBDT, XGB, LGB and CAT decreased by 38.39%, 30.08%, 27.83%, 27.43 and 26.73% respectively, compared with those of models at Harbin station. In terms of effect of input combinations on modeling accuracy, models with M5 (with RMSE ranged from 0.6494–0.7622 mm d^-1^, MAE ranged from 0.5036–0.6171 mm d^-1^, Adj_R^2^ ranged from 0.4512–0.5992) performed slightly worse than models with M3 (with RMSE ranged from 0.6322–0.7658 mm d^-1^, MAE ranged from 0.4906–0.6215 mm d^-1^, Adj_R^2^ ranged from 0.4457–0.6207), while models with M6 (with RMSE ranged from 0.6171–0.7580 mm d^-1^, MAE ranged from 0.4745–0.6138 mm d^-1^, Adj_R^2^ ranged from 0.4574–0. 6382) still performed better than models with M3, while the effect of the other input combinations was generally the same as that of Harbin station and Changsha station.

In conclusion, CAT models could offer the highest accuracy among all tested models no matter under what input combination or at which station, followed by LGB and XGB models, which could also achieve relatively satisfactory precision. There is no doubt CAT1 based on Rs obtained the best performance and be highly recommend for estimating daily ET_**0**_ in this study area. However, CAT5 and CAT6 models based on only temperature data and partial geographic data could achieve acceptable accuracy with fewest meteorological variables, which can be regarded as more cost-effective and more conducive to promotion and application.

### Comparison of model accuracy stability with different input combinations across 10 stations

**Fig 2:**
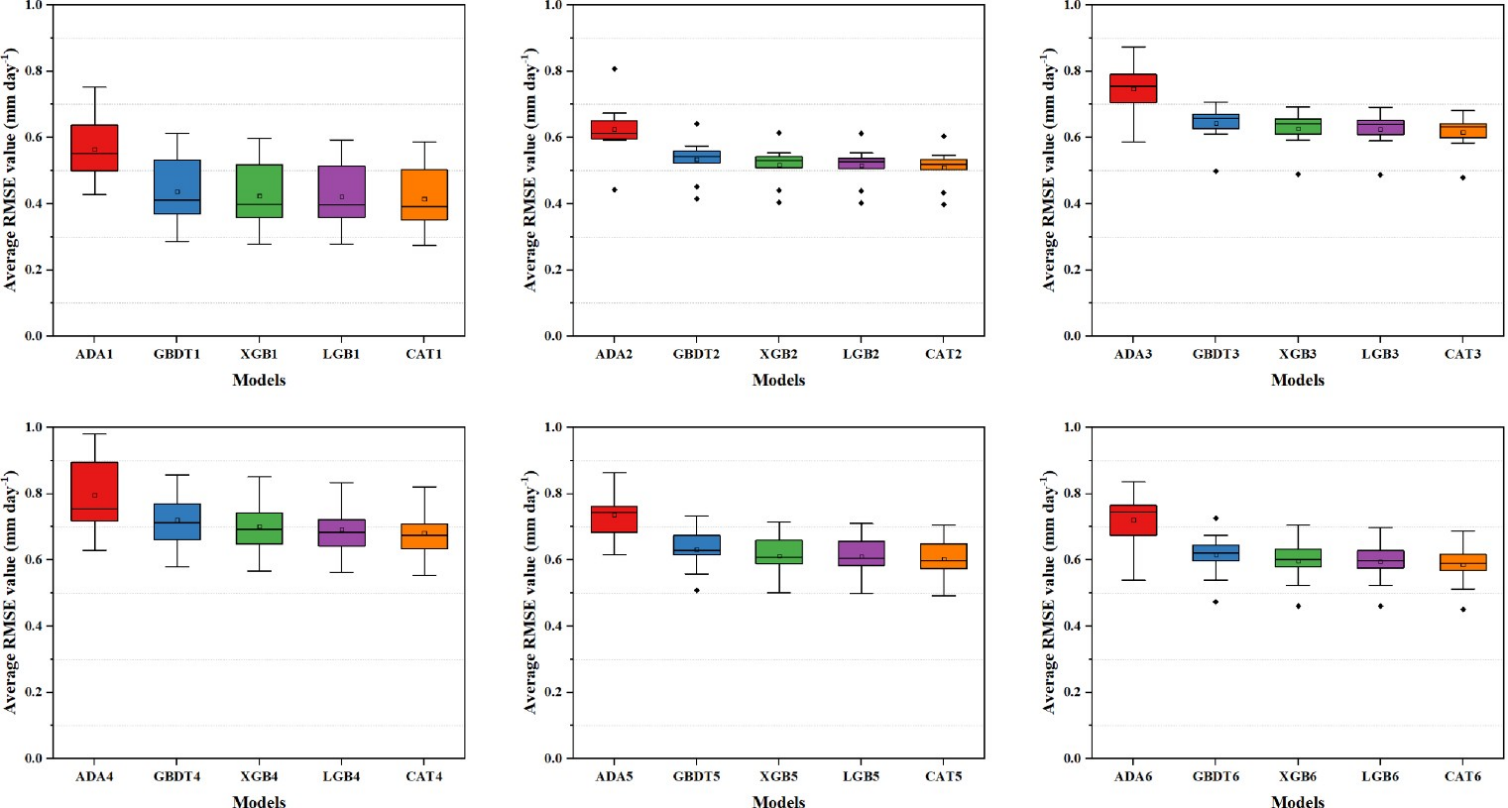
Average RMSE values of Boosting-based models at 10 stations under different input combinations.

Fig 2 demonstrated the average RMSE values of Boosting-based models with various input combinations across 10 stations as a box plot. Because of the modeling accuracy of ADA models were much worse than the other Boosting-based models, the stability comparison employed in present study mainly focuses on GBDT, XGB, LGB and CAT models. Among the tested models, CAT model not only achieved the smallest average RMSE value, but also the most concentrated distribution of RMSE values regardless of the input combinations, which indicated that the CAT model had the best precision stability. The stability of the other three models is basically the same, thus the modeling accuracy should be the primary consideration when selecting one model for estimating ET_0_ among these 3 models. In terms of the effect of input combinations, taking CAT models as example, the RMSE values of CAT model based on M2 input obtained the minimal fluctuation (with RMSE ranged from 0.4334–0.6044) across 10 stations, followed by models based on M3, M6, M5, M4 and M1. It's also worth noting that although the accuracy of models with M1 input was the highest in each station, the accuracy gap between stations across different climate zones was the largest (with RMSE ranged from 0.2746–0.5861), which may as a result of the differences in the Rs distribution among each station and the different contribution of Rs to daily ET_0_ across various climate zones.

### The results of path analysis between meteorological variables and ET_0_ at 10 stations

Path analysis is a method proposed by Sewell Wright for studying the direct and indirect effects of independent variables on dependent variables and quantitatively analyzing the mutual influence degree of factors. Therefore, this study introduced path analysis to analyze the effect of T_max_, T_min_, RH, U_2_ and Rs on daily ET_0_. The results of path analysis between meteorological variables and ET_0_ across all stations were shown in Table 13.

**Table 13:** Path analysis between meteorological variables and ET_0_ at 10 stations.

Meteorological Variables	Correlation Coefficient	Direct Effect	Indirect Effect						Contribution to R^2^
			T_max_	T_min_	RH	U_2_	R_S_	Sum	
Harbin									
T_max_	0.860	0.544	-	0.976	-0.318	-0.085	0.670	0.316	0.142
T_min_	0.787	0.003	0.976	-	-0.216	-0.097	0.567	0.784	0.000
RH	-0.614	-0.184	-0.318	-0.318	-	-0.125	-0.628	-0.430	0.026
U_2_	0.034	0.087	-0.085	-0.097	-0.125	-	-0.077	-0.053	0.007
R_S_	0.865	0.390	0.670	0.567	-0.628	-0.077	-	0.475	0.749
Shenyang									
T_max_	0.829	0.565	-	0.968	-0.016	-0.039	0.598	0.264	0.164
T_min_	0.726	0.050	0.968	-	0.130	-0.056	0.461	0.675	0.000
RH	-0.442	-0.236	-0.016	0.130	-	-0.087	-0.527	-0.205	0.038
U_2_	0.117	0.136	-0.039	-0.056	-0.087	-	-0.040	-0.019	0.018
R_S_	0.843	0.362	0.598	0.461	-0.527	-0.040	-	0.480	0.710
Yan’an									
T_max_	0.822	0.310	-	0.943	-0.156	-0.138	0.597	0.512	0.142
T_min_	0.673	0.256	0.943	-	0.068	-0.148	0.388	0.417	0.004
RH	-0.581	-0.252	-0.156	0.068	-	-0.208	-0.642	-0.330	0.033
U_2_	0.124	0.140	-0.138	-0.148	-0.208	-	0.030	-0.015	0.020
R_S_	0.870	0.420	0.597	0.388	-0.642	0.030	-	0.450	0.757
Jinan									
T_max_	0.799	0.396	-	0.946	-0.051	-0.060	0.600	0.403	0.127
T_min_	0.643	0.190	0.946	-	0.176	-0.124	0.417	0.453	0.002
RH	-0.513	-0.290	-0.051	0.176	-	-0.223	-0.532	-0.223	0.052
U_2_	0.240	0.187	-0.060	-0.124	-0.223	-	0.098	0.053	0.032
R_S_	0.856	0.366	0.600	0.417	-0.532	0.098	-	0.490	0.732
Nanjing									
T_max_	0.765	0.294	-	0.936	0.015	-0.137	0.504	0.471	0.144
T_min_	0.592	0.248	0.936	-	0.254	-0.080	0.300	0.344	0.004
RH	-0.485	-0.299	0.015	0.254	-	0.135	-0.524	-0.186	0.038
U_2_	-0.091	0.133	-0.137	-0.080	0.135	-	-0.237	-0.224	0.017
R_S_	0.866	0.519	0.504	0.300	-0.524	-0.237	-	0.347	0.750
Changsha									
T_max_	0.776	0.012	-	0.930	0.019	-0.176	0.610	0.764	0.070
T_min_	0.629	0.392	0.930	-	0.296	-0.062	0.430	0.237	0.000
RH	-0.343	-0.227	0.019	0.296	-	0.239	-0.378	-0.117	0.012
U_2_	-0.133	0.133	-0.176	-0.062	0.239	-	-0.265	-0.266	0.015
R_S_	0.927	0.701	0.610	0.430	-0.378	-0.265	-	0.226	0.859
Chengdu									
T_max_	0.793	0.260	-	0.948	-0.203	-0.002	0.595	0.532	0.104
T_min_	0.639	0.146	0.948	-	0.029	0.028	0.426	0.493	0.001
RH	-0.589	-0.229	-0.203	0.029	-	-0.115	-0.504	-0.360	0.043
U_2_	0.097	0.149	-0.002	0.028	-0.115	-	-0.140	-0.052	0.022
R_S_	0.895	0.583	0.595	0.426	-0.504	-0.140	-	0.312	0.801
Kunming									
T_max_	0.741	0.117	-	0.839	-0.368	-0.018	0.515	0.624	0.114
T_min_	0.374	0.289	0.839	-	0.102	-0.165	0.114	0.085	0.008
RH	-0.806	-0.398	-0.368	0.102	-	-0.362	-0.732	-0.408	0.060
U_2_	0.345	0.156	-0.018	-0.165	-0.362	-	0.205	0.189	0.023
R_S_	0.878	0.462	0.515	0.114	-0.732	0.205	-	0.416	0.771
Nanning									
T_max_	0.643	0.222	-	0.878	0.265	-0.306	0.587	0.421	0.042
T_min_	0.402	0.256	0.878	-	0.579	-0.263	0.409	0.146	0.006
RH	-0.367	-0.443	0.265	0.579	-	-0.220	-0.131	0.075	0.088
U_2_	-0.045	0.213	-0.306	-0.263	-0.220	-	-0.342	-0.258	0.071
R_S_	0.865	0.645	0.587	0.409	-0.131	-0.342	-	0.220	0.748
Guangzhou									
T_max_	0.527	0.104	-	0.874	0.383	-0.418	0.502	0.423	0.001
T_min_	0.270	0.348	0.874	-	0.694	-0.293	0.285	-0.078	0.069
RH	-0.349	-0.476	0.383	0.694	-	-0.127	-0.178	0.127	0.036
U_2_	-0.209	0.173	-0.418	-0.293	-0.127	-	-0.400	-0.382	0.021
R_S_	0.909	0.741	0.502	0.285	-0.178	-0.400	-	0.168	0.826

**Fig 3:**
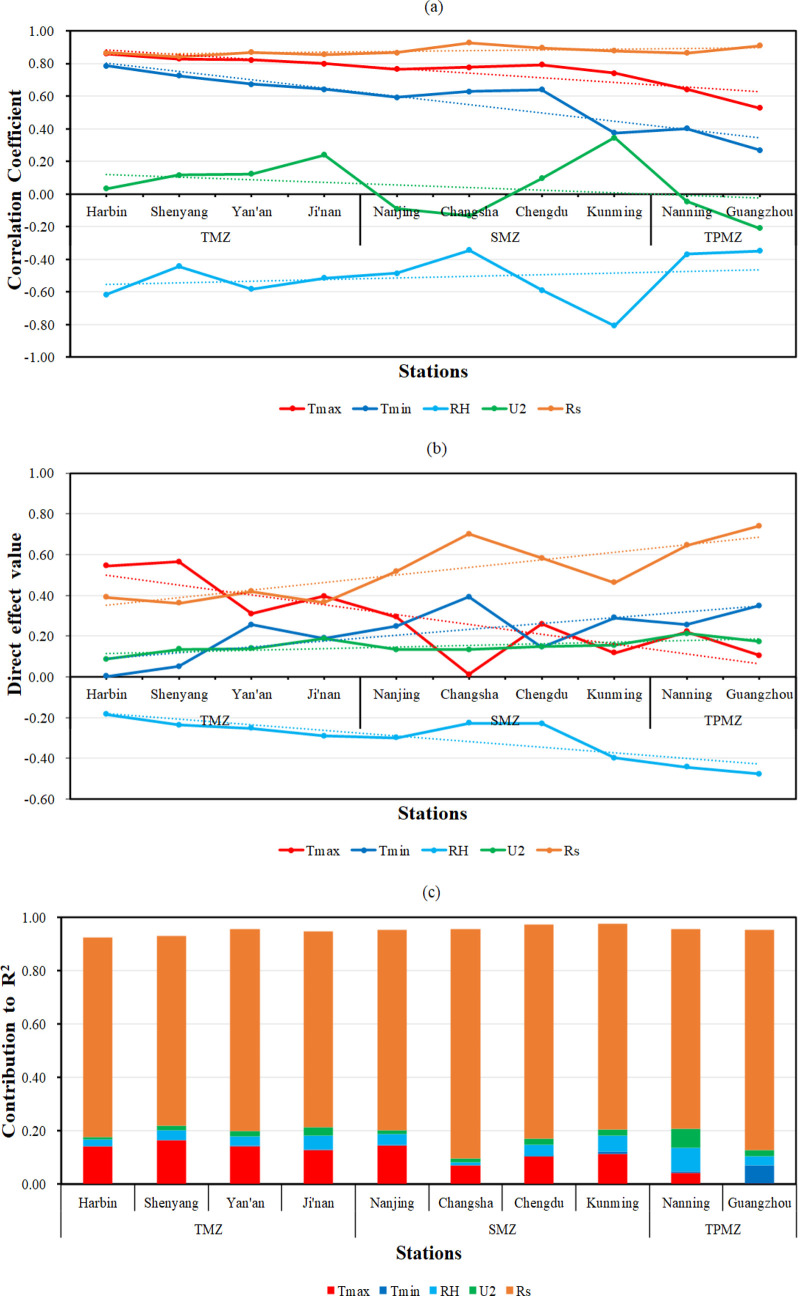
Path analysis results of meteorological variables to daily ET0 across different stations. (a) Correlation coefficient between meteorological variables and ET_0_ at 10 stations; (b) Direct effect of meteorological variables on ET_0_ at 10 stations; (c) The contribution of meteorological variables to R^2^ value at 10 stations.

It could be found from Table 13 that except for RH and U_2_, the other 3 meteorological variables all had positive correlation coefficient to ET_0_ at all 10 stations. As illustrated in Fig 3A, the dashed line is the trend line of its corresponding meteorological variables. At stations in TMZ, the correlation coefficient of T_max_, T_min_, RH, U_2_ and Rs were 0.799–0.860,0.643–0.787,-0.442- -0.614,0.034–0.240 and 0.843–0.865 respectively. When it comes to stations in SMZ, the correlation coefficient of T_max_, T_min_, RH, U_2_ and Rs were 0.741–0.793,0.374–0.639,-0.343- -0.806, -0.091–0.345 and 0.866–0.927 respectively. And the correlation coefficient of T_max_, T_min_, RH, U_2_ and Rs were 0.527–0.643,0.270–0.402,-0.349- -0.367, -0.045–0.209 and 0.865–0.909 respectively at stations in TPMZ. It’s obvious to see that from Harbin station to Guangzhou station, the correlation coefficient of T_max_, T_min_, RH and U_2_ with ET_0_ showed decrease trend in general, while only Rs were on the contrary (increased from 0.749 at Harbin station to 0.826 at Guangzhou station), which indicated that the overall contribution of Rs on ET_0_ increased significantly and became more and more crucial for accurately estimating ET_0_ as the latitude of the station goes down in this study area.

The direct effects tendency of T_max_, T_min_, RH, U_2_ and Rs on ET_0_ across 10 stations was shown in Fig 3B. At Harbin station, T_max_ contributed the largest direct effect (0.544), which was 0.154 more than Rs (0.390), but the direct effect of T_min_ was almost none, only 0.003. As the station’s latitude goes down, the direct effect of T_max_ showed a significant decrease, the direct effect of T_min_, RH (absolute value) and Rs had apparent rise, and that of U_2_ showed a slight rise. When it comes to Guangzhou station, the direct effect of T_max_ only left 0.104, while the direct effect of Rs rose to 0.711 and T_min_ exceeded T_max_ to 0.348. This trend is similarly reflected in the overall contribution of variables to R^2^. It can be seen from Fig 3C, specially, as the second most contributing variable (0.142) at Harbin station, T_max_ reduced to the least contributing variable (0.001)at Guangzhou station. On the contrary, T_min_, which is the least contributing variable at Harbin station (0.000), could offer a contribution to R^2^ of 0.069 at Guangzhou station. Other meteorological variables also gradually increase the contribution to the R^2^ of ET_0_ estimation results, as the latitude goes from north to south.

In conclusion, Rs had the greatest contribution to ET_0_, followed by T_max_, T_min_ and RH, while U_2_ generally had the least effect on daily ET_0._ These results provided an explanation for the difference in the modeling accuracy of models with same input condition between stations across different climate zones and could also offer a reliable reference for selecting appropriate input combination for ET_0_ estimation in different climate zones.

## Discussion

### Effect of Ra and J on improving model accuracy

**Fig 4:**
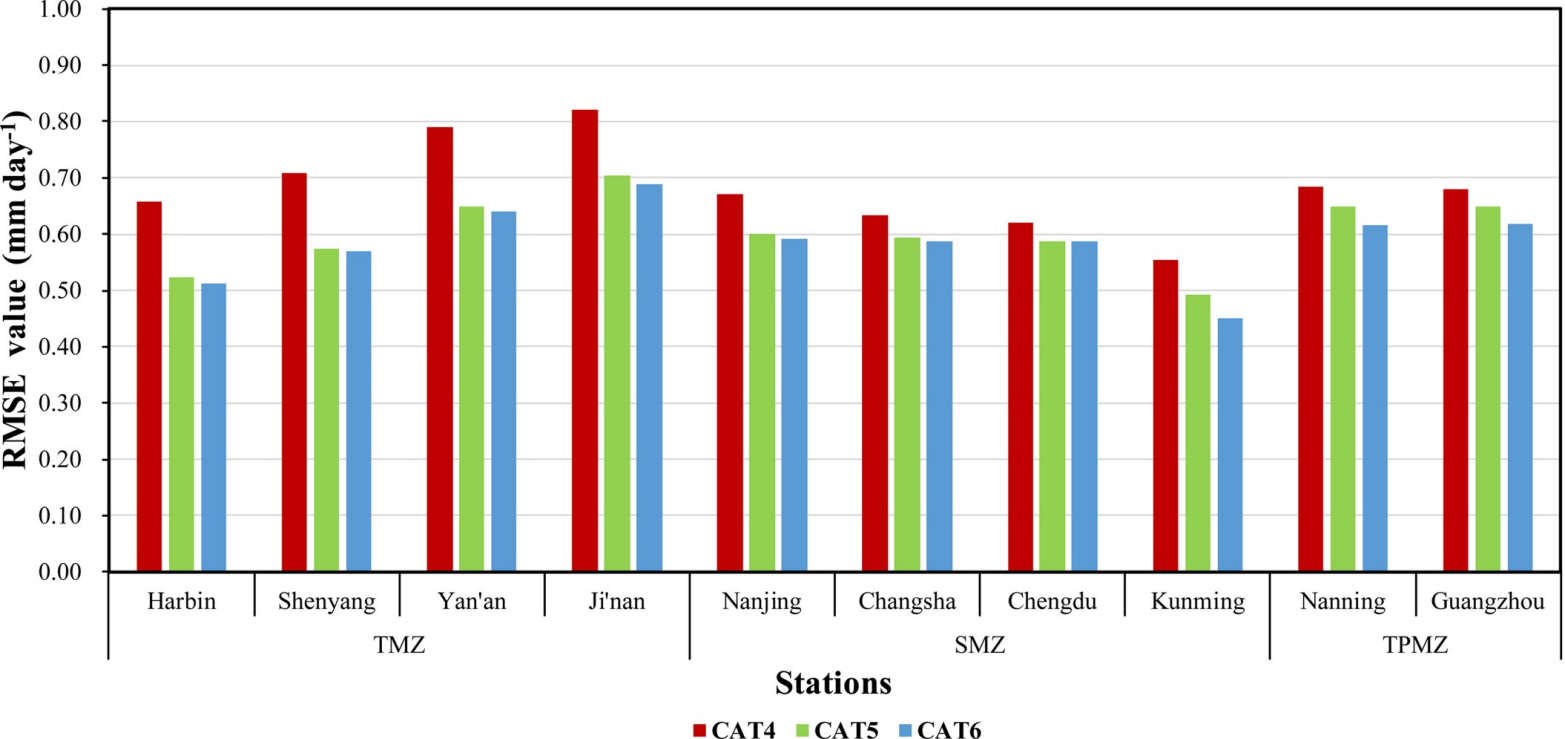
Comparison of the RMSE value of CAT4, CAT5 and CAT6 across 10 stations.

Ra has been proved that it can improve the estimation accuracy of daily ET_0_ when only limited meteorological variables are available [26,46–48]. As shown in Fig 4, taking CAT models as examples, CAT4 based on only temperature data could only obtain an average RMSE value ranging from 0.5542–0.8204 mm day^-1^, while the average RMSE values of CAT5 and CAT6 ranged from 0.4914–0.7042 mm day^-1^and 0.4507–0.6877 mm day^-1^ respectively. Compared with CAT4, employing Ra could make the average RMSE value decrease by 20.67%, 19.14%, 17.63%, 14.17%, 10.22%, 6.18%, 5.29%, 11.34%, 5.26% and 4.42% from Harbin to Guangzhou station, while using J could make that decreased by 22.22%, 19.79%, 18.93%, 16.18%, 11.72%, 7.33%, 5.10%, 18.67%, 9.95% and 9.17% respectively. It is obvious to see that CAT6 performed even better than CAT5 and this kind of improvement on modeling accuracy decreased as the station’s latitude decreases. This phenomenon may as a result of meteorological conditions of the stations in this study area are generally quite stable and ET_0_ is the result of the coupling effect of various meteorological variables, so J contains more overall information and variation pattern of ET_0_ than the single calculated Ra.

To sum up, the result of employing Ra with only temperature data for estimating ET_0_ in present study was also same as previous. As a parameter to calculate Ra, J could also improve the modeling accuracy with limited inputs and was even better than Ra. Therefore, models based on J input can be recommended for estimating ET_0_ when partial meteorological variables and geographical data are absent.

### Strategy for selecting proper input combination at different stations

According to Tables 3–12 and the results of path analysis in 3.3, we can optimize the meteorological variables involved in the input combination at different stations. For example, T_min_ has the least contribution to R^2^ and smallest direct effect on daily ET_0_ at Harbin station, thus T_min_ could be removed from these input combinations without reducing the modeling accuracy. Similarly, T_max_ could be removed from the input combinations at Guangzhou station due to the fact that it could hardly make contribution to the R^2^ value of the estimation results. The different model performance between various stations could also be explained by the path analysis results. Taking CAT models in 10-fold validation stage as examples, the correlation coefficient of RH and U2 at Kunming station are much higher than those at other stations, thus CAT2 model (with RMSE of 0.3977 mm d^-1^, MAE of 0.3106 mm d^-1^ and Adj_R^2^ of 0.9168) and CAT3 model (with RMSE of 0.4785 mm d^-1^, MAE of 0.3721 mm d^-1^ and Adj_R^2^ of 0.8618) achieved the highest precision compared with CAT2 and CAT3 models at other stations. In conclusion, the selection of the input combination for estimating daily ET_0_ should be based on the importance and contribution of meteorological variables at each single station, so as to make use of available meteorological variables more effectively to obtain better accuracy.

## Conclusion

This study investigated 5 Boosting-based models, including original Adaptive Boosting(ADA), Gradient Boosting Decision Tree(GBDT), Extreme Gradient Boosting(XGB), Light Gradient Boosting Decision Tree (LGB) and Gradient boosting with categorical features support(CAT), for accurately estimating daily ET_0_ value with 6 different meteorological variables input combinations at 10 stations across the eastern monsoon zone of China. The results indicated that the CAT models had the highest accuracy and stability over all tested models under the same input combinations across all stations. And the LGB and XGB models could achieve very close accuracy, while original ADA models produced the worst performance. Under the condition of limited meteorological variables input, Rs definitely plays the most important role for accurately estimating daily ET_0_ value which makes the models based on M1 provide the best accuracy regardless of which station. Model with M2 input combination could offer the second highest precision, while models based on M4 (only temperature data) had the worst estimation accuracy. However, when Ra and J were employed with temperature data, the modeling accuracy increased significantly. The accuracy of models based on M6 generally ranked the third place (better than models with M3 input) and models based on M5 ranked the fifth place (much better than models with M4 input). Thus, in terms of improving the accuracy of the models with limited meteorological variables, J has better effect than Ra and is more easier to obtain in this study and the improvement brought by employing J was more and more significant as the latitude of the stations increases compared with employing Ra.

In summary, the CAT could be most highly recommended for estimating daily ET_0_ and J can be highly recommended for improving the accuracy of models when limited meteorological variables are available or geographical information is absent in the eastern monsoon zone of China.

## References

[1] Allen R, Pereira L, Raes D, Smith M. Guidelines for computing crop water requirements-FAO Irrigation and drainage paper 56; FAO-Food and Agriculture Organization of the United Nations, Rome (http://www. fao. org/docrep). ARPAV (2000), La caratterizzazione climatica della Regione Veneto, Quad. Geophysics. 1998;156: 178.

[2] Antonopoulos VZ, Antonopoulos A V. Daily reference evapotranspiration estimates by artificial neural networks technique and empirical equations using limited input climate variables. Comput Electron Agric. 2017 10.1016/j.compag.2016.11.011

[3] Wu L, Fan J. Comparison of neuron-based, kernel-based, tree-based and curve-based machine learning models for predicting daily reference evapotranspiration. PLoS One. 2019 10.1371/journal.pone.0217520 31150448 PMC6544265

[4] Tabari H, Grismer ME, Trajkovic S. Comparative analysis of 31 reference evapotranspiration methods under humid conditions. Irrig Sci. 2013 10.1007/s00271-011-0295-z

[5] Gu Z, Qi Z, Burghate R, Yuan S, Jiao X, Xu J. Irrigation Scheduling Approaches and Applications: A Review. J Irrig Drain Eng. 2020 10.1061/(asce)ir.1943-4774.0001464

[6] Allen RG, Jensen ME, Wright JL, Burman RD. Operational estimates of reference evapotranspiration. Agron J. 1989;81: 650–662.

[7] Feng Y, Cui N, Gong D, Zhang Q, Zhao L. Evaluation of random forests and generalized regression neural networks for daily reference evapotranspiration modelling. Agric Water Manag. 2017 10.1016/j.agwat.2016.07.023 28154450 PMC5221669

[8] Falamarzi Y, Palizdan N, Huang YF, Lee TS. Estimating evapotranspiration from temperature and wind speed data using artificial and wavelet neural networks (WNNs). Agric Water Manag. 2014 10.1016/j.agwat.2014.03.014

[9] Xu JZ, Peng SZ, Zhang RM, Li DX. Neural network model for reference crop evapotranspiration prediction based on weather forecast. Shuili Xuebao/Journal Hydraul Eng. 2006.

[10] Khoshhal J, Mokarram M. Model for prediction of evapotranspiration using MLP neural network. Int J Environ Sci. 2012;3: 1000.

[11] Tabari H. Evaluation of reference crop evapotranspiration equations in various climates. Water Resour Manag. 2010 10.1007/s11269-009-9553-8

[12] Kisi O. Comparison of different empirical methods for estimating daily reference evapotranspiration in Mediterranean climate. J Irrig Drain Eng. 2013;140: 4013002.

[13] Shih SF. Data requirement for evapotranspiration estimation. J Irrig Drain Eng. 1984;110: 263–274.

[14] Allen RG. Self-calibrating method for estimating solar radiation from air temperature. J Hydrol Eng. 1997 10.1061/(ASCE)1084-0699(1997)2:2(56)

[15] Alexandris S, Stricevic R, Petkovic S. Comparative analysis of reference evapotranspiration from the surface of rainfed grass in central Serbia, calculated by six empirical methods against the Penman-Monteith formula. Eur Water. 2008;21: 17–28.

[16] Jensen M, Haise H. Estimating Evapotranspiration from Solar Radiation. Proc Am Soc Civ Eng J Irrig Drain Div. 1963.

[17] Hargreaves GH, Samani ZA. Reference crop evapotranspiration from temperature. Appl Eng Agric. 1985;1: 96–99.

[18] PRIESTLEY CHB, TAYLOR RJ. On the Assessment of Surface Heat Flux and Evaporation Using Large-Scale Parameters. Mon Weather Rev. 1972 10.1175/1520-0493(1972)100<0081:otaosh>2.3.co;2

[19] Irmak S, Irmak A, Allen RG, Jones JW. Solar and net radiation-based equations to estimate reference evapotranspiration in humid climates. J Irrig Drain Eng. 2003 10.1061/(ASCE)0733-9437(2003)129:5(336)

[20] Makkink GF. Testing the Penman formula by means of lysismeters. Int Water Eng. 1957.

[21] Kumar M, Raghuwanshi NS, Singh R, Wallender WW, Pruitt WO. Estimating evapotranspiration using artificial neural network. J Irrig Drain Eng. 2002 10.1061/(ASCE)0733-9437(2002)128:4(224)

[22] Abdullah SS, Malek MA, Abdullah NS, Kisi O, Yap KS. Extreme Learning Machines: A new approach for prediction of reference evapotranspiration. J Hydrol. 2015;527: 184–195. 10.1016/j.jhydrol.2015.04.073

[23] Kişi Ö. Evapotranspiration modeling using a wavelet regression model. Irrig Sci. 2011 10.1007/s00271-010-0232-6

[24] Feng Y, Cui N, Zhao L, Hu X, Gong D. Comparison of ELM, GANN, WNN and empirical models for estimating reference evapotranspiration in humid region of Southwest China. J Hydrol. 2016 10.1016/j.jhydrol.2016.02.053

[25] Zheng H, Yuan J, Chen L. Short-Term Load Forecasting Using EMD-LSTM neural networks with a xgboost algorithm for feature importance evaluation. Energies. 2017 10.3390/en10081168

[26] Fan J, Yue W, Wu L, Zhang F, Cai H, Wang X, et al Evaluation of SVM, ELM and four tree-based ensemble models for predicting daily reference evapotranspiration using limited meteorological data in different climates of China. Agric For Meteorol. 2018 10.1016/j.agrformet.2018.08.019

[27] Kisi O. Modeling reference evapotranspiration using three different heuristic regression approaches. Agric Water Manag. 2016 10.1016/j.agwat.2016.05.010

[28] Kişi O, Çimen M. Evapotranspiration modelling using support vector machines. Hydrol Sci J. 2009 10.1623/hysj.54.5.918

[29] Wu L, Huang G, Fan J, Zhang F, Wang X, Zeng W. Potential of kernel-based nonlinear extension of Arps decline model and gradient boosting with categorical features support for predicting daily global solar radiation in humid regions. Energy Convers Manag. 2019 10.1016/j.enconman.2018.12.103

[30] Malik A, Kumar A, Kisi O. Monthly pan-evaporation estimation in Indian central Himalayas using different heuristic approaches and climate based models. Comput Electron Agric. 2017;143: 302–313. 10.1016/j.compag.2017.11.008

[31] Shiri J, Nazemi AH, Sadraddini AA, Landeras G, Kisi O, Fakheri Fard A, et al Comparison of heuristic and empirical approaches for estimating reference evapotranspiration from limited inputs in Iran. Comput Electron Agric. 2014 10.1016/j.compag.2014.08.007

[32] Mosavi A, Edalatifar M. A Hybrid Neuro-Fuzzy Algorithm for Prediction of Reference Evapotranspiration. Lecture Notes in Networks and Systems. 2019 10.1007/978-3-319-99834-3_31

[33] Mohammadi B, Mehdizadeh S. Modeling daily reference evapotranspiration via a novel approach based on support vector regression coupled with whale optimization algorithm. Agric Water Manag. 2020 10.1016/j.agwat.2020.106145

[34] Breiman L. Random forests. Mach Learn. 2001 10.1023/A:1010933404324

[35] Friedman JH. Greedy function approximation: A gradient boosting machine. Ann Stat. 2001 10.2307/2699986

[36] Chen T, Guestrin C. Xgboost: A scalable tree boosting system. Proceedings of the 22nd acm sigkdd international conference on knowledge discovery and data mining. ACM; 2016. pp. 785–794.

[37] Wang S, Fu Z, Chen H, Ding Y, Wu L, Wang K. Simulation of Reference Evapotranspiration Based on Random Forest Method. Nongye Jixie Xuebao/Transactions Chinese Soc Agric Mach. 2017 10.6041/j.issn.1000-1298.2017.03.038

[38] Huang G, Wu L, Ma X, Zhang W, Fan J, Yu X, et al Evaluation of CatBoost method for prediction of reference evapotranspiration in humid regions. J Hydrol. 2019 10.1016/j.jhydrol.2019.04.085

[39] Fan J, Ma X, Wu L, Zhang F, Yu X, Zeng W. Light Gradient Boosting Machine: An efficient soft computing model for estimating daily reference evapotranspiration with local and external meteorological data. Agric Water Manag. 2019 10.1016/j.agwat.2019.05.038

[40] Allen RG, Pereira LS, Raes D, Smith M. Crop evapotranspiration-Guidelines for computing crop water requirements-FAO Irrigation and drainage paper 56. Fao, Rome. 1998;300: D05109.

[41] Freund Y, Schapire R, Abe N. A short introduction to boosting. Journal-Japanese Soc Artif Intell. 1999;14: 1612.

[42] Freund Y, Schapire RE. A Decision-Theoretic Generalization of On-Line Learning and an Application to Boosting. J Comput Syst Sci. 1997 10.1006/jcss.1997.1504

[43] Ke G, Meng Q, Finley T, Wang T, Chen W, Ma W, et al LightGBM: A highly efficient gradient boosting decision tree. Adv Neural Inf Process Syst. 2017;2017–Decem: 3147–3155.

[44] Prokhorenkova L, Gusev G, Vorobev A, Dorogush AV, Gulin A. Catboost: Unbiased boosting with categorical features. Advances in Neural Information Processing Systems. 2018.

[45] Landeras G, Ortiz-Barredo A, López JJ. Comparison of artificial neural network models and empirical and semi-empirical equations for daily reference evapotranspiration estimation in the Basque Country (Northern Spain). Agric water Manag. 2008;95: 553–565.

[46] Valiantzas JD. Temperature-and humidity-based simplified Penman’s ET0 formulae. Comparisons with temperature-based Hargreaves-Samani and other methodologies. Agric Water Manag. 2018 10.1016/j.agwat.2018.06.028

[47] Yu T, Cui N, Zhang Q, Hu X. Applicability evaluation of daily reference crop evapotranspiration models in Northwest China. Paiguan Jixie Gongcheng Xuebao/Journal Drain Irrig Mach Eng. 2019 10.3969/j.issn.1674-8530.18.0256

[48] Feng Y, Peng Y, Cui N, Gong D, Zhang K. Modeling reference evapotranspiration using extreme learning machine and generalized regression neural network only with temperature data. Comput Electron Agric. 2017 10.1016/j.compag.2017.01.027

